# Bis(bicyclo[1.1.1]pentyl)chlorophosphine as a Precursor for the Preparation of Bis(bicyclo[1.1.1]pentyl)phosphines

**DOI:** 10.1021/acs.orglett.4c01190

**Published:** 2024-05-12

**Authors:** Griffin L. Perry, Nathan D. Schley

**Affiliations:** Department of Chemistry, Vanderbilt University, Nashville, Tennessee 37235, United States

## Abstract

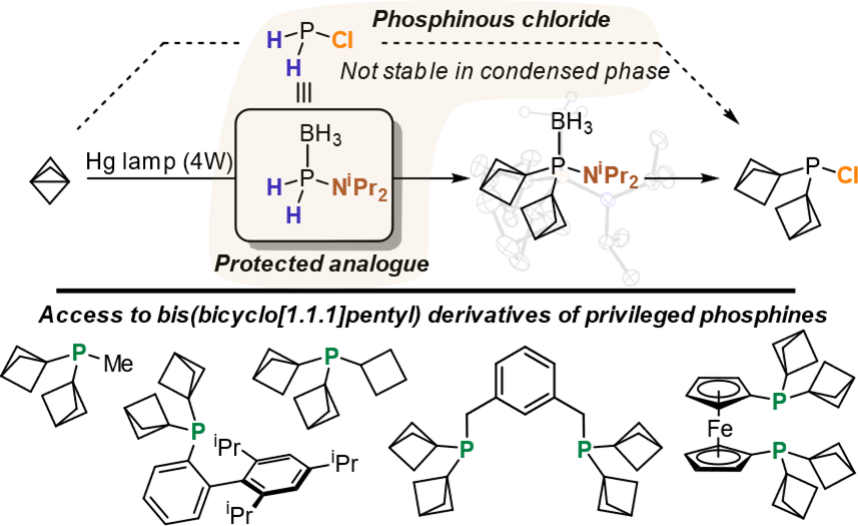

Dialkylchlorophosphines are among the most versatile building blocks for tertiary phosphine ligands, but their synthesis relies on the nucleophilic substitution of PCl_3_, leaving substituents that require P–H precursors largely inaccessible. The primary phosphine reagent iPr_2_NPH_2_·BH_3_ can serve as a doubly protected PH_2_Cl proxy, enabling the synthesis of bis(bicyclo[1.1.1]pentyl)chlorophosphine (Bcp_2_PCl) for the first time. Bcp_2_PCl serves as a general reagent for the preparation of a family of bis(bicyclo[1.1.1]pentyl) alkyl- and arylphosphines, including new members of privileged phosphine ligand scaffolds.

**Introduction.** Organophosphines are among the most commonly employed ligands in organometallic chemistry, contributing to the efficacy of nearly every major class of transformations performed with homogeneous catalysis. For many transformations, so-called “privileged” ligand classes have emerged that tend to confer either exceptional catalyst performance or unusual substrate generality.^1^ Since minor modifications to a ligand’s structure can dramatically impact reactivity in ways that are difficult to predict,^2^ the availability of privileged ligand classes for a transformation can be viewed as necessary to the wide adoption of that transformation by the synthetic chemistry community. Most privileged phosphine ligands follow the general form R_2_PR′ with R being a common substituent (e.g., ^i^Pr, Ph, ^t^Bu, Cy) and R′ being the distinguishing fragment. Dialkylbiarylphosphines developed by Buchwald are a canonical example, as modification of the ligand’s biaryl or dialkyl substituents has led to highly effective catalysts, often optimized for a particular substrate class.^1b^ Many privileged diphosphines follow a similar formula including ferrocenyl diphosphines^3^ (e.g., dppf, MandyPhos,^4^ and Josiphos^5^ ligands), binap,^6^ spirobiindane,^7^ and SEGPHOS^8^ derivatives. In nearly all cases, the final phosphine is synthesized by nucleophilic addition to an electrophilic dialkylchlorophosphine derivative. Thus, much of the expansive literature on dialkylphosphines depends on the availability of suitable dialkylchlorophosphine building blocks for the continued development of new and effective catalytic systems.

In a recent report we described the synthesis and applications of tris(bicyclo[1.1.1]pentyl)phosphine (PBcp_3_) in palladium-catalyzed sp^3^-electrophile cross-coupling reactions.^2c^ The bicyclo[1.1.1]pentyl group was first introduced in organophosphorus chemistry with Wiberg’s synthesis of Ph_2_P(Bcp) in 1990^9^ but has remained largely unexplored as a substituent in phosphine ligands for transition metal complexes. The lack of a direct route to bicyclo[1.1.1]pentyllithium or Grignard reagents leaves radical addition of P–H bonds across the reactive C–C bond of [1.1.1]propellane as the most suitable route for bicyclo[1.1.1]pentyl phosphine synthesis.^2c,9,10^ Indeed, our synthesis of PBcp_3_ relied on the radical alkylation of PH_3_. Adapting this route to an expansive collection of bis(bicyclo[1.1.1]pentyl) derivatives of privileged phosphine scaffolds would require the synthesis of the corresponding primary phosphine in each case. Very few primary phosphines are commercially available owing to their stench and extreme oxygen sensitivity.^8^ Still, our observation that bicyclo[1.1.1]pentyl substituents impart desirable properties of stability, crystallinity, compact steric profile, and electron donor power to the resulting ligand^2c^ have encouraged a search for an alternative, convergent strategy.

## Results and Discussion.

 Since the majority of organophosphine synthetic routes to privileged phosphines rely on the availability of dialkylchlorophosphines, we aimed to develop a synthetic route to chlorobis(bicyclo[1.1.1]pentyl)phosphine (Bcp_2_PCl). As radical addition of a P–H bond to [1.1.1]propellane currently represents the most direct means of introducing the bicyclo[1.1.1]pentyl moiety, we canvassed the literature for suitable phosphorus starting materials. Phosphinous chloride (PH_2_Cl) has no appreciable lifetime in the condensed phase,^11^ likely stemming from the incompatibility of chlorophosphines with primary or secondary phosphine P–H bonds. For instance, PPh_2_Cl reacts with PPh_2_H to give tetraphenyldiphosphine^12^ and treatment of PH_3_ with dilute chlorine gives P_2_H_4_.^13^ At the same time, there are very few protected primary phosphorus(III) derivatives available with the exception of Me_3_SiPH_2_, whose synthesis begins at PH_3_ via the corresponding phosphide.^14^ PH_3_’s toxicity and flammability are manageable, especially on small scales,^2c,15^ but we posited that its use here would limit the number of researchers willing to explore bis(bicyclo[1.1.1]pentyl)phosphine derivatives and so have endeavored to find an alternate strategy.

**Figure 1:**
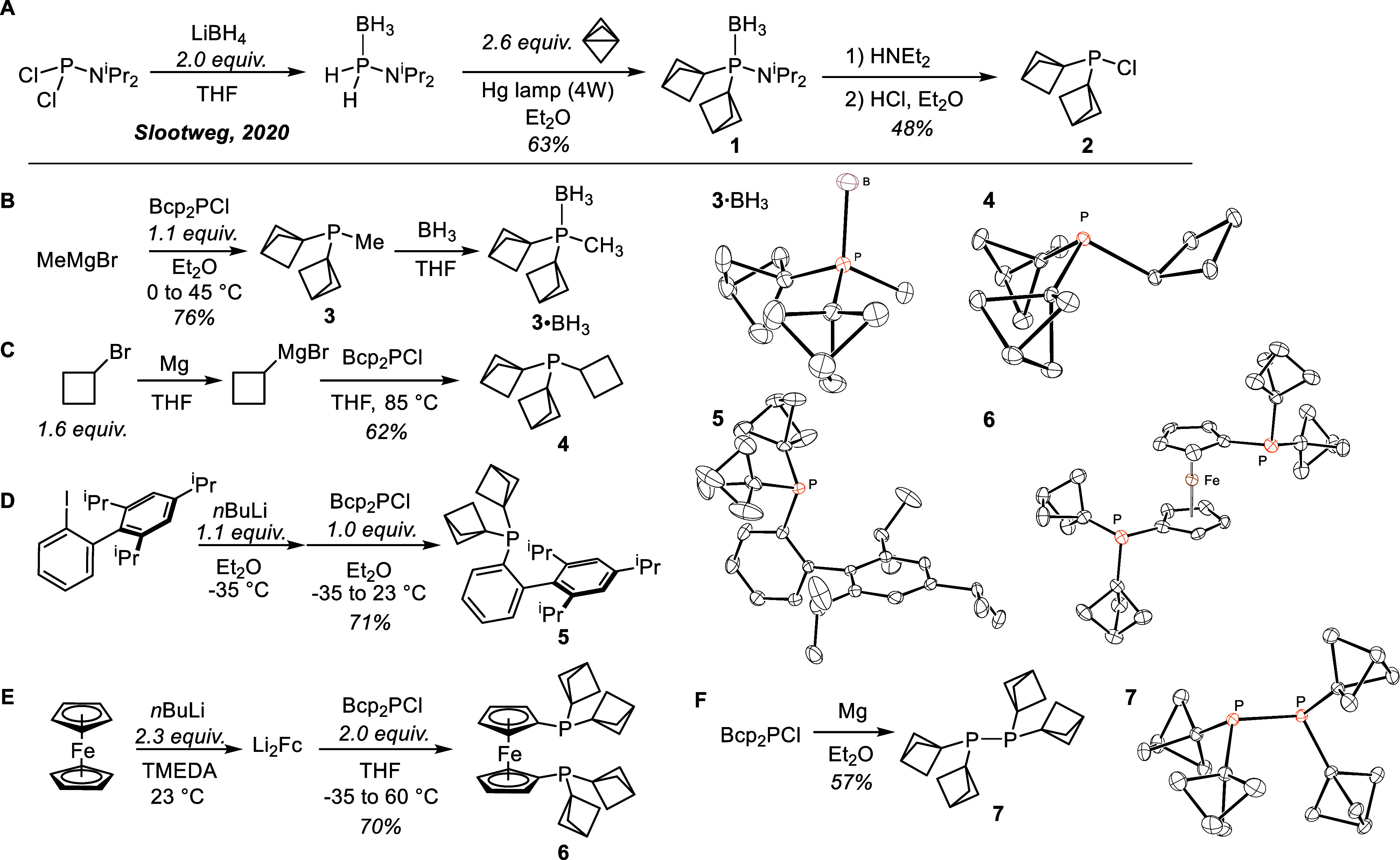
A) Synthesis of Bcp_2_PCl (**2**) from Slootweg’s iPr_2_NPH_2_·BH_3_. B–E) Applications of **2** in the synthesis of tertiary phosphines. F) Reduction of **2** to the diphosphine. Right: ORTEPs of **3**·BH_3_, **4**, **5**, **6**, and **7** are shown at 50% probability.

Recently, Slootweg and co-workers described the synthesis of a series of primary amidophosphine boranes (RPH_2_·BH_3_) via reduction of the corresponding amidodichlorophosphine with lithium borohydride.^16^ By this method, they were able to prepare iPr_2_NPH_2_·BH_3_, which we posited could serve as a rare example of a stable PH_2_Cl synthon. To our delight, iPr_2_NPH_2_·BH_3_ undergoes UV-promoted alkylation by [1.1.1]propellane to give the (diisopropylamido)bis(bicyclo[1.1.1]pentyl)phosphine borane (Bcp_2_PN^i^Pr_2_·BH_3_) (**1**) as a stable, colorless solid (Figure 1, A).

Bcp_2_PN^i^Pr_2_·BH_3_ can be converted into the desired chlorophosphine through a two-step sequence. Borane deprotection with diethylamine gives the putative amidodialkylphosphine Bcp_2_PN^i^Pr_2_, which can be chlorinated directly without isolation using ethereal HCl (Figure 1, A).^17^ The chlorophosphine Bcp_2_PCl (**2**) is conveniently purified by Kugelrohr distillation to give the product as a colorless oil. Thus, Bcp_2_PCl is accessible in four steps from commercially available compound iPrN_2_PCl or five steps from PCl_3_. This route avoids the need for PH_3_ and validates Slootweg’s amidophosphine borane iPr_2_NPH_2_·BH_3_ as a convenient PH_2_Cl synthon.

With a suitable and scalable route to Bcp_2_PCl in hand, we set about exploring its versatility as a building block for the synthesis of an array of organophosphorus compounds. Treatment of Bcp_2_PCl with MeMgBr gives the tertiary phosphine Bcp_2_PMe (**3**) as a colorless oil (Figure 1, part B). This straightforward procedure exemplifies the necessity of our having developed a route to Bcp_2_PCl, since synthesis of Bcp_2_PMe would have otherwise required radical alkylation of MePH_2_ with propellane. MePH_2_ is a pyrophoric gas and its protected analogue MePH_2_·BH_3_ is a volatile liquid.^18^ Bcp_2_PMe can be conveniently converted to a crystalline solid by protection as the BH_3_ adduct. Secondary alkyl Grignards are also compatible reagents, as in the reaction of cyclobutyl magnesium bromide with Bcp_2_PCl to give cyclobutyl bis(bicyclo[1.1.1]pentyl)phosphine (Bcp_2_PCyb, **4**) as a colorless solid (Figure 1, C). Related trialkylphosphines can be highly effective ligands for enabling difficult oxidative addition reactions at Pd.^2b,2c^ The Bcp group has in common with tBu rigorous 3-fold symmetry and a lack of conformational flexibility that few other substituents possess. Computational predictions^19^ of steric and electronic Tolman parameters^20^ place Bcp_2_PCyb (**4**) (163.2°, 2058.6 cm^–1^) as similar in size and donor power to P^i^Pr_3_ (160.0°, 2059.2 cm^–1^). Bcp_2_PMe (**3**) (158.2°, 2061.3 cm^–1^) is smaller and slightly less donating than the related phosphine ^t^Bu_2_PMe (164.2°, 2059),^21^ being closer to PEt_3_ (132.0°, 2061.7 cm^–1^) in donor power.^2c,20^

Bis(bicyclo[1.1.1]pentyl) arylphosphines are also accessible from Bcp_2_PCl. Lithiation of *o*-(2,4,6-triisopropylphenyl)phenyl iodide followed by treatment with Bcp_2_PCl gives **5**, the bis(bicyclo[1.1.1]pentyl) analogue of XPhos (Figure 1, D).^22^ Related dialkylbiarylphosphine ligands have been effectively utilized in a variety of cross-couplings and aminations.^1b^ Bcp_2_PCl can also be used in the preparation of bidentate bis(bicyclo[1.1.1]pentyl) derivatives. For example, treatment of Li_2_Fc·TMEDA with Bcp_2_PCl provides **6**, the bicyclopentyl analogue of dppf, (Bcp_2_P)_2_Fc (Figure 1, E). Dppf variants are commonly applied in Ni- and Pd-catalyzed transformations and are generally considered a privileged ligand class.^1c,23^ The properties of (Bcp_2_P)_2_Fc are being explored in ongoing efforts.

Bcp_2_PCl also has potential applications beyond tertiary phosphine synthesis. Treatment with magnesium turnings^12d^ gives the diphosphine Bcp_4_P_2_ (**7**) as the exclusive product without evidence for reductive C–C or C–P bond scission of the strained carbocyclic skeleton (Figure 1, F). Analogous diphosphines are useful reagents in diphosphination reactions.^12c,24^

**Figure 2:**
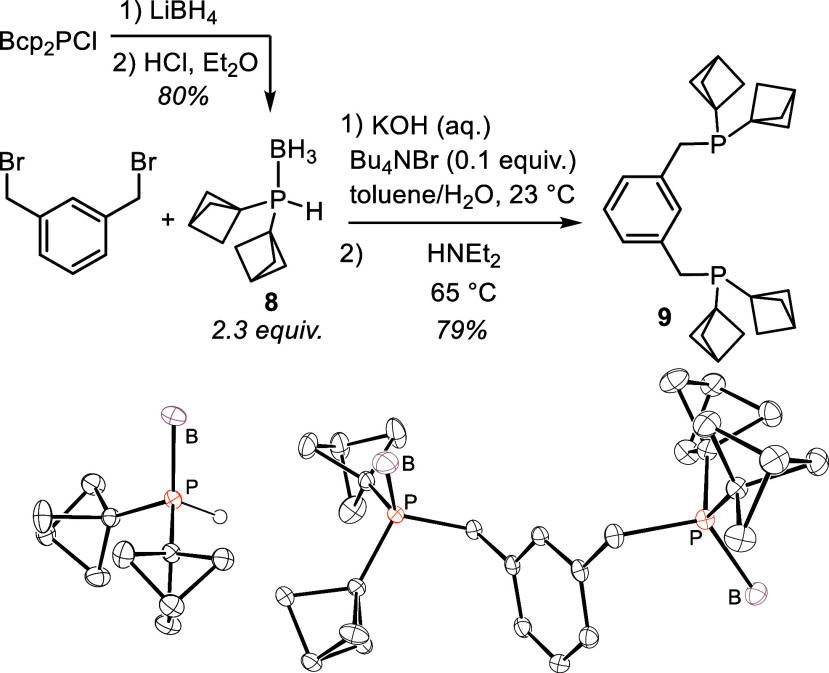
Synthesis and an application of the protected 2° phosphine Bcp_2_PH·BH_3_ (**8**), (top). ORTEPs of **8** (bottom left) and **9**·2BH_3_ (bottom right) are shown at 50% probability.

Bcp_2_PCl also undergoes reduction with LiBH_4_ to give the corresponding borane-protected secondary phosphine Bcp_2_PH·BH_3_ (**8**) (Figure 2). In our previous report^2c^ we showed that alkylation of PH_3_ with [1.1.1]propellane proceeds to PBcp_3_ even in the presence of excess PH_3_; therefore, reduction of Bcp_2_PCl provides the only current means to access this protected secondary phosphine. Secondary phosphines are more air-sensitive than their corresponding chlorophosphines and have a disagreeable odor, but these downsides are tempered by protection of the borane adduct. We have successfully employed Bcp_2_PH·BH_3_ in the preparation of the so-called PCP pincer ligand ^Bcp4^PCP (**9**). Under phase-transfer conditions^25^ Bcp_2_PH·BH_3_ serves as a nucleophilic phosphine reagent, which gives ^Bcp4^PCP after deprotection. This procedure gave the highest yields of several we explored (Figure 2).

## Conclusions.

 Slootweg’s phosphine reagent iPr_2_NPH_2_·BH_3_ serves as a doubly protected proxy for phosphinous chloride (PH_2_Cl), a species that is inaccessible in the condensed phase. Radical addition of [1.1.1]propellane followed by halogenation gives bis(bicyclo[1.1.1]pentyl)chlorophosphine. This precursor is easily prepared on gram scales and provides straightforward access to a host of bis(bicyclo[1.1.1]pentyl) alkyl- and arylphosphines, including members of privileged phosphine ligand scaffolds. We expect that this route will improve the availability of Bcp phosphine derivatives in catalytic studies and will provide a route to other chlorophosphines that would benefit from a PH_2_Cl synthon, for instance, those derived of olefin hydrophosphination.^26^ Our ongoing work aims to more broadly explore the applications of bis(bicyclo[1.1.1]pentyl)phosphine ligands in catalysis.

## Data Availability

The data underlying this study are available in the published article and its Supporting Information.

## Supporting Information Available

Experimental procedures, summaries of computational and experimental results, compound characterization data, NMR and IR spectra, and X-ray crystallographic data (PDF)
Atomic coordinates (XYZ)

## Supplementary Material

ol4c01190_si_001.pdf

ol4c01190_si_002.xyz
