# Supplementary material for: Bis(bicyclo[1.1.1]pentyl)chlorophosphine as a Precursor for the Preparation of Bis(bicyclo[1.1.1]pentyl)phosphines
Source: Org Lett. 2024 May 12;26(29):6071–5. doi: 10.1021/acs.orglett.4c01190 (PMC11287751; doi:10.1021/acs.orglett.4c01190)
Supplement: Supplementary file 1 — ol4c01190_si_001.pdf [file ol4c01190_si_001.pdf]

Supporting Information

Bis(bicyclo[1.1.1]pentyl)chlorophosphine as a Precursor for the Preparation of Bis(bicyclo[1.1.1]pentyl)phosphines

Griffin L. Perry and Nathan D. Schley*

Department of Chemistry, Vanderbilt University, Nashville, Tennessee 37235 United States

**I.**	**General Information**	**1**
**II.**	**Synthesis and Characterization**	**2**
**III.**	**Computational Methods**	**10**
**IV.**	**Spectra**	**11**
**V.**	**X-ray Crystallographic Data**	**41**
**VI.**	**References**	**58**

I. General Information

General Considerations. All manipulations were carried out using standard vacuum, Schlenk, cannula, or glovebox techniques under N_2 unless otherwise specified. Tetrahydrofuran, dichloromethane, pentane, toluene and diethyl ether were degassed with argon and dried over activated alumina using a solvent purification system. $HNEt_2$ was dried over 3Å molecular sieves for at least one day before use. All other reagents are broadly available and were used as received. Quartz round bottom flasks were obtained from Technical Glass Products, Inc.

Spectroscopy. ^1H, $^{13}C\{^1H\}$ and $^{31}P\{^1H\}$ NMR spectra were recorded on Bruker NMR spectrometers at ambient temperature unless otherwise noted. ^1H and $^{13}C\{^1H\}$ chemical shifts are referenced to residual solvent signals; $^{31}P\{^1H\}$ chemical shifts are referenced to an external H_3PO_4 standard. FT-IR spectra were collected on a Thermo Nicolet IR100 spectrometer with a diamond ATR accessory.

Mass Spectrometry. High resolution mass spectrometry was conducted by the Mass Spectrometry Research Center (MSRC) at Vanderbilt University. Solutions of purified products were diluted into an acid-free carrier solvent and analyzed in positive mode by ESI using an Orbitrap mass analyzer. Satisfactory analyses were not obtained for the more-sensitive compounds **2** and **8** however, their characterization is unambiguous since derivatives thereof were completely characterized by NMR, X-ray, and HRMS methods.

II. Synthesis and Characterization

Diisopropylamido phosphine borane ($iPr_2NPH_2·BH_3$). This compound was prepared as previously described.[1] Note: This procedure can be scaled up successfully to at least 3x the reported scale with comparable yield. The product can be obtained by concentration to an oil as described, or alternatively, the concentration of the pentane solution can be assayed by quantitative NMR against an internal standard. On occasion the LiCl byproduct is incompletely removed by the single filtration reported. In such cases, a precipitate may be observed when preparing dilute solutions in pentane like those used in the synthesis of bis(bicyclo[1.1.1]pentyl)diisopropylamidophosphine borane (*vide infra*). A transparent solution is important for UV-promoted alkylation, thus if a pentane solution of $iPr_2NPH_2·BH_3$ appears turbid, it should be filtered prior to use (e.g. through a 0.45 μm PTFE syringe filter).

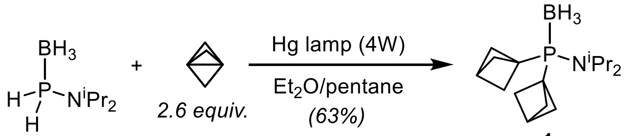

Bis(bicyclo[1.1.1]pentyl)diisopropylamido phosphine borane ($Bcp_2PN^iPr_2·BH_3$) (1):

Preparation of the [1.1.1]-propellane solution. A solution of propellane in Et_2O was freshly prepared using phenyllithum according to a reported procedure.[2] After short-path distillation, the solution was dried over anhydrous $MgSO_4$ and degassed by 2 freeze-pump-thaw cycles. The concentration was assayed by ^1H NMR using dichloroethane as an internal standard in analogy to a reported procedure,[3] and the propellane solution stored at –35 °C until ready for use.

Preparation of $Bcp_2PN^iPr_2·BH_3$ A solution of $iPr_2NPH_2·BH_3$ in pentane[1] (15.89 mmol, 96.9 mM in 164 mL) was added to a 500 mL quartz round bottom flask under nitrogen. The solution was illuminated with a 4W 254 nm UV lamp placed directly against the flask, and a propellane solution (105 mL, 0.397 M in Et_2O, 41.7 mmol, 2.62 equiv.) was added over 10 minutes. Stirring and illumination were continued for 8.5 h, then the round bottom was stored in a -20 °C freezer until workup the following morning (12 h). The solution was concentrated *in vacuo*, then, in a glovebox, the oily residue was extracted with three, 12 mL portions of pentane, filtered through a 0.45 μm PTFE syringe filter, and the extracts combined and concentrated. 5 mL of n-heptane was added to the tacky residue, and the mixture stored in a -35 °C freezer overnight. The contents were filtered to collect the solid which was dried, ground with a spatula, and dried under vacuum to give the product as a colorless solid. Yield: 2.8 g, (63 %). Crystals used for X-ray analysis were grown by cooling a saturated pentane solution in a -35° freezer.

^{1}H NMR (400 MHz, $CDCl_3$): δ 3.36 (dq, 2H, J = 14.8, 6.8 Hz), 2.67 (d, 2H, $^{4}J_{H-P}$ = 40.0 Hz), 2.10 (ap. q, 18H), 1.25 (d, 12H, J = 6.8 Hz)

$^{13}C\{^{1}H\}$ NMR (151 MHz, $CDCl_3$): δ 52.6 (s, Bcp), 48.5 (s, $((CH_3)2C-H)$, 41.7 (d, 27 Hz, Bcp), 32.5 (d, 27 Hz, Bcp), 24.1 (s, ^{i}Pr)

$^{31}P\{^{1}H\}$ NMR (162 MHz, $CDCl_3$): δ 45.26 (m)

HRMS (ESI) m/z $[M+H]^+$ calcd for $C_{16}H_{31}BNPH^+$: 280.2360; found: 280.2367

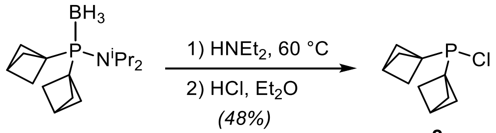

Bis(bicyclo[1.1.1]pentyl)phosphine chloride (Bcp_2PCl) (2): In an inert atmosphere glove box a 25 mL pressure flask was charged with 3.3 g of bis(bicyclo[1.1.1]pentyl) diisopropylamido phosphine borane ($Bcp_2PN^iPr_2{\cdot}BH_3$) and 10 mL of dry $HNEt_2$. The vessel was sealed and heated in a 60 °C oil bath for 18 hours. The resulting solution was evaporated under vacuum to an oil and 10 mL of pentane was then added and the solution filtered through oven-dried alumina. The alumina was rinsed with an additional 20 mL of pentane and the combined filtrate was concentrated to *ca.* 4 mL at which point the solution became turbid. The residue was filtered through a 0.45 μm PTFE syringe filter and was then thoroughly concentrated under vacuum.

The resulting oil was diluted with 10 mL of pentane and added to a 100 mL pressure flask. 60 mL of a 1 M HCl solution in diethyl ether (5 equiv.) was then added slowly. The flask was sealed, and the turbid mixture stirred at room temperature for 16 hours. The solution was concentrated under vacuum and the resulting residue was extracted with four, 5 mL portions of pentane. The combined extracts were filtered through a 0.45 μm PTFE syringe filter and were concentrated under vacuum into a small round bottom flask in preparation for distillation in a Kugelrohr apparatus. Kugelrohr distillation was performed at 30 - 35 °C and 0.2 torr to give the product as a colorless oil in the second bulb. Yield: 1.128 g, (47.6 %)

^{1}H NMR (400 MHz, $CDCl_3$): δ 2.77 (d, 2H, $^{4}J_{H-P}$ = 29.5 Hz), 2.00 (s, 12H)

$^{13}C\{^{1}H\}$ NMR (101 MHz, $CDCl_3$): δ 51.3 (d, $^{3}J_{C-P}$ = 8.2 Hz), 44.4 (d, $^{1}J_{C-P}$ = 57.5 Hz), 32.8 (d, $^{2}J_{C-P}$ = 25.6 Hz)

$^{31}P\{^{1}H\}$ NMR (162 MHz, $CDCl_3$): δ 82.2

^{31}P NMR (162 MHz, $CDCl_3$): δ 82.17, (d, $^{4}J_{P-H}$ = 29.4 Hz)

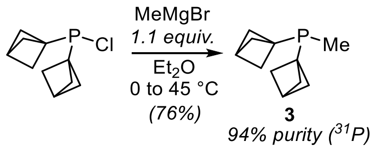

Bis(bicyclo[1.1.1]pentyl)methylphosphine (Bcp_2PMe) (3). In an inert atmosphere glove box a 25 mL pressure tube was charged with a solution of Bcp_2PCl (0.0928 g, 0.46 mmol) in 6 mL Et_2O. The flask was cooled to -35 °C in a freezer for 10 minutes, then removed and treated with 0.16 mL of a 3M MeMgBr solution (1.1 equiv.) added dropwise. The tube was sealed, removed from the glovebox, and stirred in an ice bath at 0 °C. The flask was allowed to come to room temperature over the course of 1.5 hours, at which point the flask was heated at 45 °C for 1.5 hours, cooled to room temperature, and treated with 6 mL of H_2O. The aqueous layer was extracted with five, 5 mL portions of Et_2O and the combined organic extracts were dried over $MgSO_4$ and filtered through a funnel to give a turbid oil. Pentane (3 mL) was added and the mixture stored in a -35 °C freezer for 10 minutes. The oil was filtered through a 0.45 μm PTFE syringe filter. The residue in the vial was washed with 3 mL pentane, stored at -35 °C for 10 minutes, and filtered, and the combined filtrates were concentrated under vacuum to give the product as a colorless oil. The phosphine obtained by this method was 94% pure by ^{31}P NMR, with the balance suspected to be Bcp_2PH based on its chemical shift and similar boiling point. Yield: 0.0671 g, (75.7% based on 94% purity). Single crystals for X-ray crystallography were obtained after conversion to the borane adduct with BH_3·THF. Crystals were grown by cooling a saturated pentane solution in a -35° freezer.

^{1}H NMR (400 MHz, C_6D_6): δ 2.62 (d, 2H, 25.6 Hz), 1.79 (m, 12H), 0.80 (m, 3H)

$^{13}C\{^{1}H\}$ NMR (101 MHz, C_6D_6): δ 51.3 (d, 6.8 Hz), 43.5 (d, 36.2 Hz, Bcp), 32.9 (d, 23.3 Hz, Bcp), 6.4 (d, 13.6 Hz, Me)

$^{31}P\{^{1}H\}$ NMR (162 MHz, C_6D_6): δ -48.3

HRMS (ESI) m/z $[M+H]^+$ calcd for $C_{11}H_{17}PH^+$: 181.1141; found: 181.1151

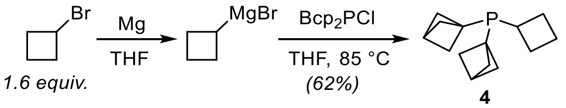

Bis(bicyclo[1.1.1]pentyl)cyclobutylphosphine (Bcp_2PCyb) (4): Magnesium turnings were ground to a powder with a mortar and pestle, 0.045 g of which were added to a Schlenk flask with 30 mL THF. A single crystal of iodine was added, and the suspension stirred with gentle heating with a heat gun until the solution became colorless. Cyclobutyl bromide (0.10 mL, 1.1 mmol) was added slowly, and the mixture stirred for 2 hours at room temperature. The solution was then decanted off the excess magnesium via cannula into a pressure tube containing a solution of Bcp_2PCl (0.1372 g, 0.68 mmol) in 5 mL THF. The tube was sealed and heated in an 85 °C oil bath overnight. Then 6 mL of degassed

H_2O was added, and the organic layer was separated. The aqueous layer was extracted with five, 5 mL portions of Et_2O and the combined extracts were dried over $MgSO_4$. The supernatant was decanted and the $MgSO_4$ washed with 2 mL Et_2O. The combined solutions were filtered through a 0.45 μm PTFE syringe filter and concentrated under vacuum to give the product as a spectroscopically pure oil. The oil was treated with 2 mL of pentane and the mixture placed in a -35 °C freezer until colorless crystals deposited. The crystals were collected on a filter funnel, dried, and were ground to give the product as a tacky solid. Yield: 0.0933 g, (61.9%) Single crystals for X-ray crystallography were obtained by cooling a saturated pentane solution in a -35° freezer.

^1H NMR (400 MHz, C_6D_6): δ 2.61 (d, 2H, $^4J_{H\text{-}P}$ = 24.5 Hz, Bcp), 2.42 (m, 1H, Cyb), 1.98-2.11 (m, 6H, Cyb), 1.83 (m, 12H, Bcp)

$^{13}C\{^1H\}$ NMR (101 MHz, C_6D_6): δ 52.7 (d, 6.3 Hz), 42.7 (d, 38.5 Hz), 33.8 (d, 21.8 Hz), 30.9 (d, 12.8 Hz, Cyb), 27.1 (d, 11.3 Hz, Cyb CH_2), 22.1 (d, 11.0 Hz, Cyb)

$^{31}P\{^1H\}$ NMR (162 MHz, C_6D_6): δ -25.0

HRMS (ESI) m/z $[M+H]^+$ calcd for $C_{14}H_{21}PH^+$: 221.1454; found: 221.1463

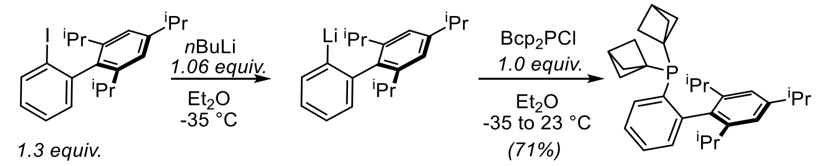

5

BcpXPhos (5): In an inert atmosphere glovebox, 0.1504 g (0.37 mmol, 1.3 equiv.) of iodo(triisopropyl)biphenyl[4] was dissolved in 5 mL Et_2O in a 20 mL vial. The solution was cooled in a -35 °C freezer, then a solution of n-butyllithium (0.12 mL of 2.5 M solution, 0.30 mmol, 1.06 equiv.) was added slowly to the chilled biaryl solution. The vial was capped and returned to the freezer for 30 minutes. A second vial was prepared containing a solution of Bcp_2PCl (0.0567 g, 0.28 mmol, 1.0 equiv.) in 2 mL Et_2O which was also chilled to -35 °C. At this point the cold solution of Bcp_2PCl was added to the cold aryl lithium solution with the aid of 1 mL Et_2O, and the resulting suspension was allowed to warm to room temperature with stirring. The vial was stirred for 18.5 hours, then, the mixture was concentrated under vacuum to a residue, which was extracted with three, 20 mL portions of pentane. The combined extracts were filtered through a 0.45 μm PTFE syringe filter and were then concentrated to 2 mL under vacuum and purified by silica gel chromatography (0-5% EtOAc/hexanes) in air. Fractions containing the product were collected and concentrated to give a colorless solid. This material was further purified by fractional recrystallization of a saturated pentane solution cooled to -35 °C, giving colorless crystals. The supernatant was decanted with a pipette and the crystals were dried to give the product as a colorless solid. The filtrate was concentrated and cooled to yield a second crop of colorless crystals. Both vials of crystals were washed twice with 1

mL portions of cold n-heptane and dried. The combined solids (crop 1: 0.0246 g, crop 2: 0.0645 g) were identical and analytically pure by ^{31}P and ^{1}H NMR spectroscopy. Overall Yield: 0.0891 g, (70.9%). Single crystals for X-ray crystallography were obtained by cooling a saturated pentane solution in a -35° freezer.

^{1}H NMR (400 MHz, CD_2Cl_2) δ 7.71 (dt, *J* = 7.4, 2.3 Hz, 1H), 7.37 (m, 2H), 7.07 (m 1H), 7.02 (s, 2H), 2.95 (hept, *J* = 6.9 Hz, 1H), 2.68 (d, *J* = 27.2 Hz, 2H), 2.36 (hept, *J* = 6.8 Hz, 2H), 1.93 (m, 12H), 1.31 (d, *J* = 6.9 Hz, 6H), 1.15 (d, *J* = 6.9 Hz, 6H), 0.93 (d, *J* = 6.8 Hz, 6H).

$^{13}C\{^{1}H\}$ NMR (151 MHz, C_6D_6) δ 148.3, 148.2 (d, *J* = 35.6 Hz), 146.6, 137.9 (d, *J* = 6.1 Hz), 136.5 (d, *J* = 13.2 Hz), 134.7 (d, *J* = 4.1 Hz), 131.8 (d, *J* = 6.8 Hz), 128.7, 126.4, 120.5, 53.0 (d, *J* = 8.6 Hz), 43.7 (d, *J* = 41.4 Hz), 34.8, 33.6 (d, *J* = 23.3 Hz), 30.9, 26.0, 24.4, 23.7.

$^{31}P\{^{1}H\}$ NMR (162 MHz, CD_2Cl_2): δ -40.5

HRMS (ESI) m/z $[M+H]^+$ calcd for $C_{31}H_{41}PH^+$: 445.3019; found: 445.3022

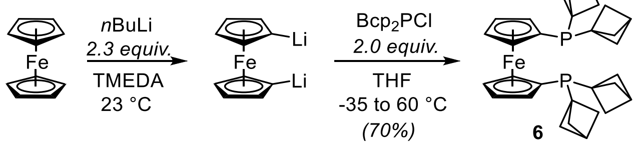

($Bcp_2P)_2Fc$ (6): In an inert atmosphere glove box, a 25 mL pressure flask was charged with a stir bar, ferrocene (0.121 g, 0.65 mmol), 5 mL pentane, and tetramethylethylene diamine (0.225 mL, 1.50 mmol). This solution was treated with 0.6 mL of a 2.5 M n-butyllithium solution (1.5 mmol). The flask was then sealed and stirred at room temperature for 16 hours. The resulting suspension was filtered to give an orange precipitate which was washed with 2 mL pentane and 2 mL Et_2O successively. The dilithioferrocene (0.1218 g, 0.47 mmol assuming the mono-TMEDA adduct) was dried under vacuum and used without further purification.

A solution of Bcp_2PCl (0.1546 g, 0.77 mmol) in 3 mL THF was chilled in a -35 °C freezer and was treated dropwise with a 10 mL n-heptane of the crude dilithioferrocene. The mixture was warmed to room temperature and stirred for 1 hour, then heated in a 60 °C oil bath for 19 hours. The crude material was concentrated under vacuum, then filtered through silica. The product was eluted with a 40% ethyl acetate/hexanes solution and dried under vacuum to give the product as an orange solid. Yield: 0.139 g, (70.1%). Single crystals for X-ray crystallography were obtained by cooling a saturated pentane solution in a -35° freezer.

^{1}H NMR (400 MHz, C_6D_6): δ 4.25 (dt, J = 7.3, 1.8 Hz, 4H), 2.63 (d, J = 26.8 Hz, 2H), 1.83 – 2.07 (m, 12H)

$^{13}C\{^{1}H\}$ NMR (101 MHz, C_6D_6): δ 76.9 (d, J = 11.6 Hz), 72.4 (d, J = 12.6 Hz), 71.6, 53.1 (d, J = 7.5 Hz), 43.4 (d, J = 37.6 Hz), 33.7 (d, J = 23.6 Hz)

$^{31}P\{^{1}H\}$ NMR (162 MHz, C_6D_6): δ -34.1

HRMS (ESI) m/z $[M+H]^+$ calcd for $C_{30}H_{36}FeP_2H^+$: 515.1709; found: 515.1698

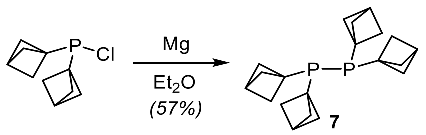

tetrakis(bicyclo[1.1.1]pentyl)diphosphine (Bcp_4P_2) (7): In an inert atmosphere glove box a 20 mL vial was charged with Bcp_2PCl (72.9 mg, 0.363 mmol), 5 mL Et_2O and a stir bar. The vial was placed in a -35 °C freezer in a glovebox for 10 minutes, then removed from the freezer and solid magnesium powder (0.0047 g, 0.53 equiv., 0.193 mmol) was added. The suspension was stirred for 17 hours at room temperature, then filtered through a 0.45 µm PTFE syringe filter and the reaction assayed by ^{31}P NMR. The crude ^{31}P NMR spectrum indicated a 15:85 ratio of product to starting material (-32 ppm : +80 ppm). The solution was returned to the freezer to chill for 10 minutes and was then removed and treated with a second aliquot of magnesium powder (0.029 g, 3.3 equiv., 1.2 mmol). The suspension was stirred for 17 hours, then filtered through a 0.45 µm PTFE syringe filter, and rinsed with 3 mL of pentane. ^{31}P NMR analysis of the combined solution indicated complete reduction, thus the solution was concentrated under vacuum to give the product as a spectroscopically pure colorless solid. Yield: 0.0342 g, (57%). Single crystals for X-ray crystallography were obtained by cooling a saturated pentane solution in a -35° freezer.

^1H NMR (400 MHz, C_6D_6): δ 2.65 (virt. q, 4H), 2.03 (virt. q, 24H)

$^{13}C\{^1H\}$ NMR (101 MHz, C_6D_6): δ 54.8 (t, J = 5.3 Hz), 41.4 (t, J = 19.4 Hz), 34.0 (t, J = 11.6 Hz)

$^{31}P\{^1H\}$ NMR (162 MHz, C_6D_6): -32.5

HRMS (ESI) m/z $[M+H]^+$ calcd for $C_{20}H_{28}P_2H^+$: 331.1739; *not observed*.
HRMS (ESI) m/z $[M+O+H]^+$ calcd for $C_{20}H_{28}P_2OH^+$: 347.1688; found: 347.1684.
Observation of the diphosphine monooxide via HRMS likely stems from the sensitivity of the compound with respect to oxidation.

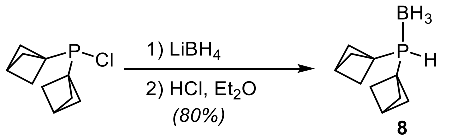

Bis(bicyclo[1.1.1]pentyl)phosphine borane ($Bcp_2PH{\cdot}BH_3$) (8): In an inert atmosphere glove box a 20 mL vial was charged with a stir bar and 0.15 mL of a commercial 4M $LiBH_4$ Et_2O solution (0.6 mmol) along with 5 mL of Et_2O. The vial was chilled in a -35 °C freezer for 10 minutes, then removed from the freezer and treated dropwise with a solution of Bcp_2PCl (0.1158 g, 0.577 mmol) in 3 mL Et_2O. The resulting suspension was stirred for 3 hours at room temperature at which point 3 mL of n-heptane was added and the vial

placed in a -35 °C freezer for 14 hours to aid precipitation. The suspension was then filtered through a 0.45 μm PTFE syringe filter and the vial washed with an additional 3 mL of pentane. The combined clear filtrate was concentrated to *ca.* 4 mL at which point it became turbid. It was filtered again, and the residue was washed with an additional 3 mL of pentane. The combined filtrates were concentrated to give the product as an oil which solidified on standing in the freezer. Yield: 0.083 g, (79.9%). Single crystals for X-ray crystallography were obtained by cooling a saturated pentane solution in a -35° freezer.

^{1}H NMR (400 MHz, C_6D_6): δ 4.19 (dq, J = 355.7, 7.2 Hz, 1H), 2.34 (d, J = 40.0 Hz, 2H), 1.78 (ap. q, J = 1.7 Hz, 12H)

^{13}C{^{1}H} NMR (151 MHz, C_6D_6): δ 52.3, 35.6 (d, J = 18.5 Hz), 34.1 (d, J = 28.0 Hz)

^{31}P{^{1}H} NMR (162 MHz, C_6D_6): -15.3 (q, $^{1}J_{P\text{-}B}$ = 40 Hz)

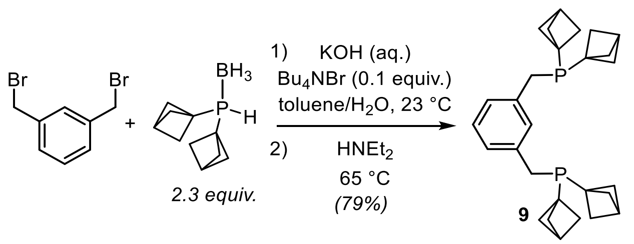

^{Bcp4}PCP (9): A 100 mL Schlenk flask was charged with bis(bicyclo[1.1.1]pentyl)phosphine borane (0.3349 g, 1.86 mmol, 2.23 equiv.) and Bu_4NBr (0.0544 g, 0.2 equiv.) in 10 mL toluene under nitrogen. To this flask was added 35 mL of an N_2-sparged 30% aqueous KOH solution. The mixture was stirred for 2 minutes, then cooled to 0 °C in an ice bath. 1,3-bis(bromomethyl)benzene (0.2201 g, 0.834 mmol, 1 equiv.) in 3 mL toluene was added slowly to the cooled mixture. The ice bath was then removed, and the mixture stirred for 16 hours. In air, the aqueous layer was extracted with six, 100 mL portions of Et_2O. The combined organic fractions were washed sequentially with 30 mL H_2O and 30 mL brine. The solution was then dried over $MgSO_4$, filtered and concentrated under vacuum to give a colorless solid.

This solid was added to a pressure flask along with 8 mL $HNEt_2$. The turbid mixture dissolved upon heating in a 65 °C oil bath. The solution was stirred at 65 °C for 18 hours, then evaporated under vacuum to give a tacky solid. This solid was dissolved in 12 mL pentane and filtered through a 0.45 μm PTFE syringe filter followed by filtration through a short pad of silica. The silica plug was washed with 8 mL pentane and the combined filtrate was dried to a tacky solid. This residue was washed with two, 3 mL portions of H_2O to remove residual amine-borane. The resulting colorless solid was dried under vacuum to give the product. Yield: 0.2866 g, (79.1%) Single crystals for X-ray crystallography were obtained after conversion to the bis(borane) adduct with $BH_3{\cdot}THF$. Crystals were grown by cooling a saturated pentane solution in a -35° freezer.

^{1}H NMR (600 MHz, CD_2Cl_2): δ 7.18 (s, 1H), 7.10 (t, J = 7.5 Hz, 1H7.18 (s, 1H), 7.03 (d, J = 7.6 Hz, 2H), 2.72 (d, J = 2.6 Hz, 4H), 2.68 (d, J = 25.9 Hz, 4H), 1.81 – 1.91 (m, 24H)

$^{13}C\{^{1}H\}$ NMR (151 MHz, CD_2Cl_2): δ 139.6 (d, J = 7.6 Hz), 130.8 (t, J = 7.6 Hz), 128.3, 127.0 (dd, J = 7.2, 2.4 Hz), 52.2 (d, J = 6.5 Hz), 43.0 (d, J = 38.7 Hz), 33.5 (d, J = 22.7 Hz), 31.6 (d, J = 12.8 Hz)

$^{31}P\{^{1}H\}$ NMR (162 MHz, C_6D_6): δ -30.0

HRMS (ESI) m/z $[M+H]^+$ calcd for $C_{28}H_{36}P_2H^+$: 435.2365; found: 435.2340

III. Computational Methods

General Methods. Density functional theory (DFT) calculations were performed using Gaussian 16.[5] The supplemental file "calc_coords.xyz" contains the computed Cartesian coordinates of the molecules computed in this study.

Prediction of Tolman electronic parameters for Bcp_2PCyb and Bcp_2PMe:
Following the procedure reported by Gusev and coworkers[6] a DFT optimization and frequency calculation was performed for $(Bcp_2PCyb)Ni(CO)_3$ and $Bcp_2PMeNi(CO)_3$ using the MPW1PW91 functional[7-9] with the following basis sets: (CHOP: 6-311+g(d,p), Ni: 6-311+g(2d)). The computed A_1 frequencies of 2157.9 cm^{-1} and 2160.7 cm^{-1} give predicted TEPs of 2058.6 cm^{-1} and 2061.30 cm^{-1} for Bcp_2PCyb and Bcp_2PMe respectively after application of the reported scaling factor of 0.9540.[6]

Computation of Tolman cone angle for Bcp_2PCyb and Bcp_2PMe: The optimized coordinates from the electronic parameters calculations above were used as input coordinates for the cone angle calculations. Tolman cone angles were calculated using the FindSolidAngle package for Mathematica.[10]

Table S1. Comparison of ligand cone angles and Tolman parameters.

Ligand	Cone Angle	TEP (cm^{-1})
P^tBu_3	182° [a]	2056 [a]
PCy_3	170° [a]	2056 [a]
$PBcp_3$	167° [c]	2061 [c]
P^iPr_3	160° [a]	2059 [a]
Bcp_2PCyb (**4**)	163° [d]	2059 [d]
P^tBu_2Me	164° [e]	2059 [b]
Bcp_2PMe (**3**)	158° [d]	2061 [d]
BcpXPhos (**5**)	-	2059 [d]
PPh_3	145° [a]	2069 [a]

a. Value from reference 11. These TEP values are experimental.
b. Computed value from reference 12.
c. Experimental value from reference 13.
d. Computed here.
e. Computed value from reference 14.

Table S2. Summary of computed energies and frequencies

Molecule	Free energy (a.u.)	imaginary freq.
Bcp_2PMe (**3**)	-2618.873431	none
Bcp_2PCyb (**4**)	-2735.510812	none
BcpXPhos (**5**)	-3395.106328	none

IV. Spectra

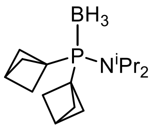

1

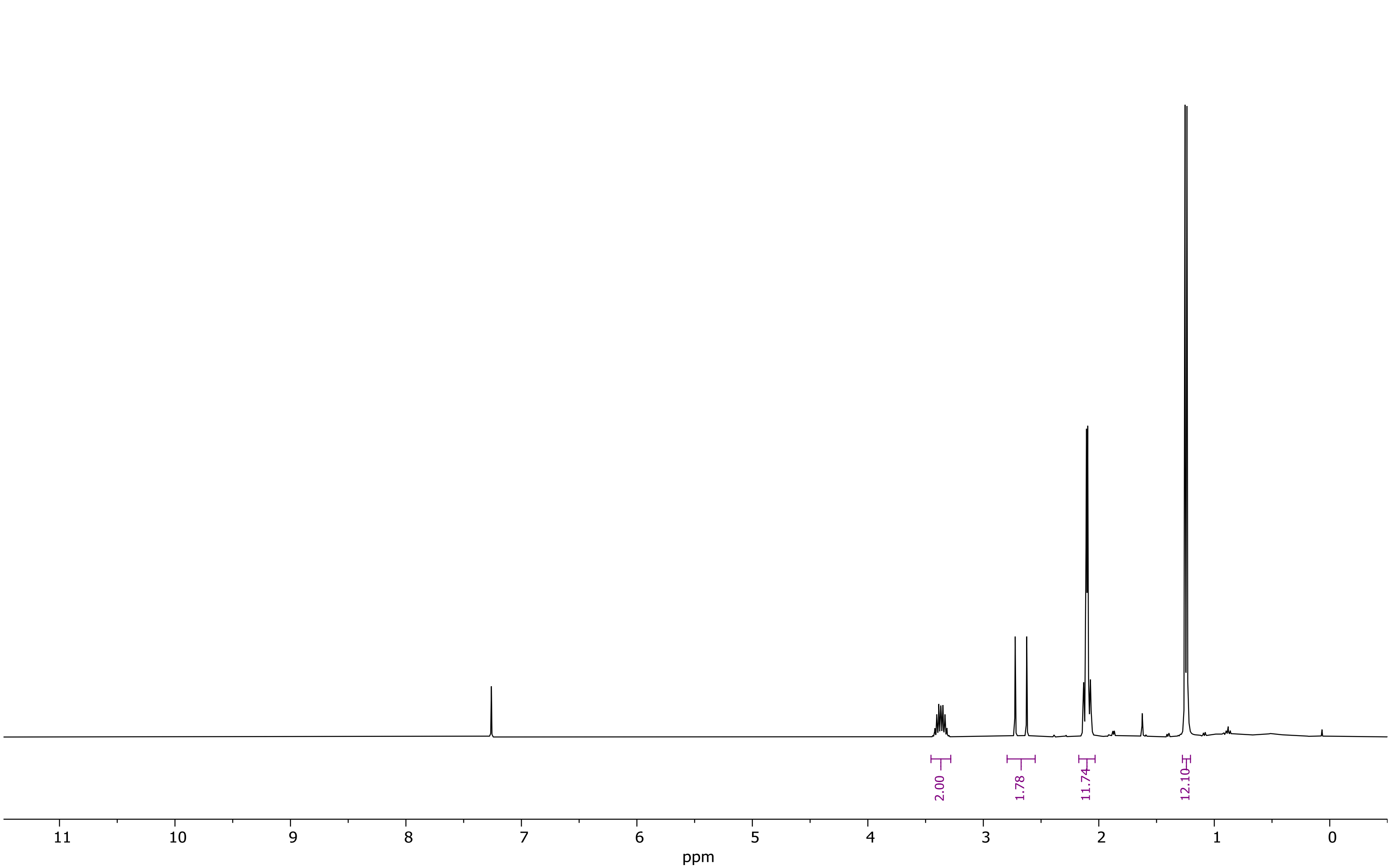

Figure S1. ^{1}H NMR Spectrum of $Bcp_2PN^iPr_2{\cdot}BH_3$ (**1**) (400 MHz, $CDCl_3$).

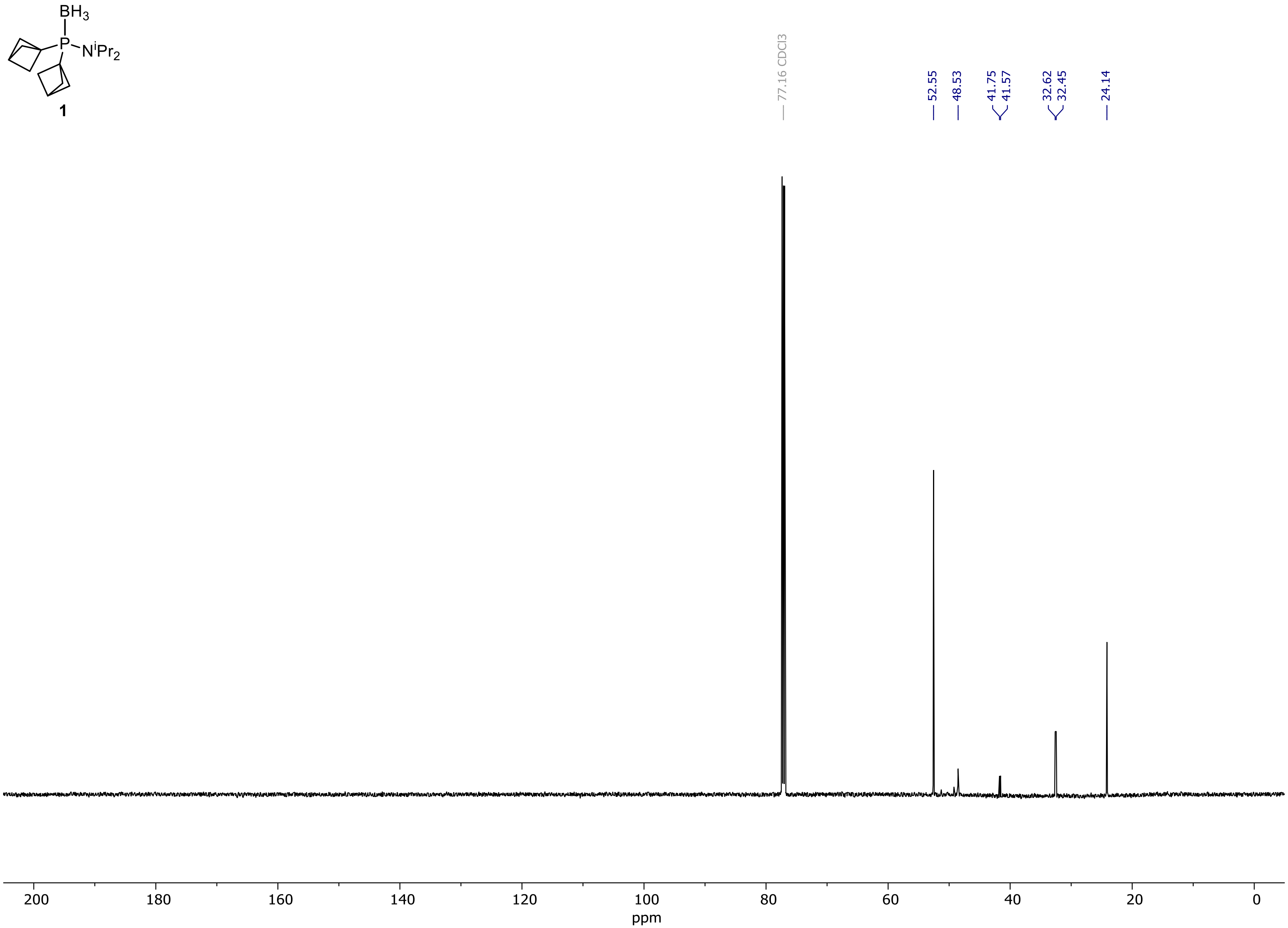

Figure S2. $^{13}C\{^1H\}$ NMR Spectrum of $Bcp_2PN^iPr_2{\cdot}BH_3$ (**1**) (151 MHz, $CDCl_3$).

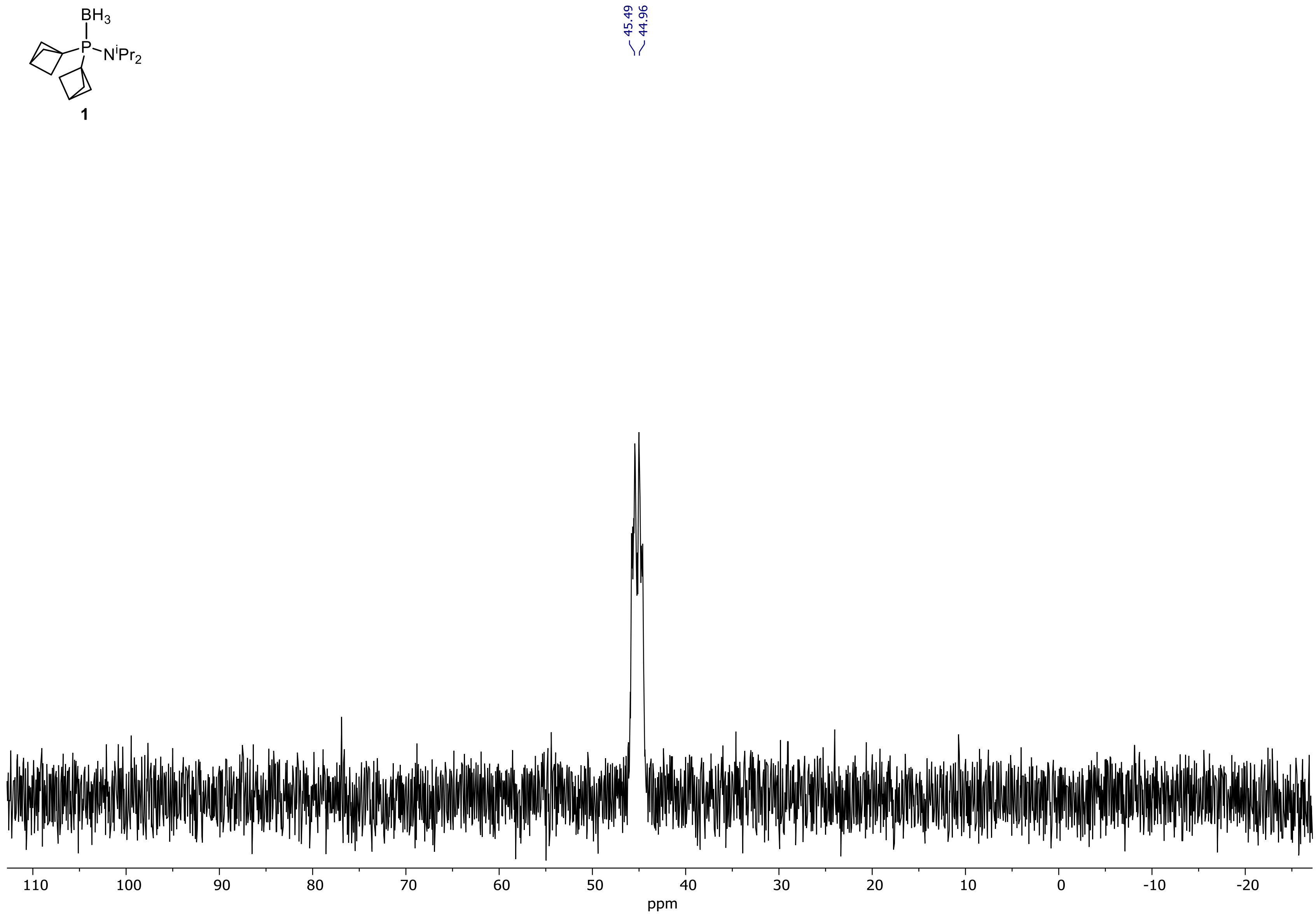

Figure S3. $^{31}P\{^1H\}$ NMR Spectrum of $Bcp_2PN^iPr_2{\cdot}BH_3$ (**1**) (162 MHz, $CDCl_3$).

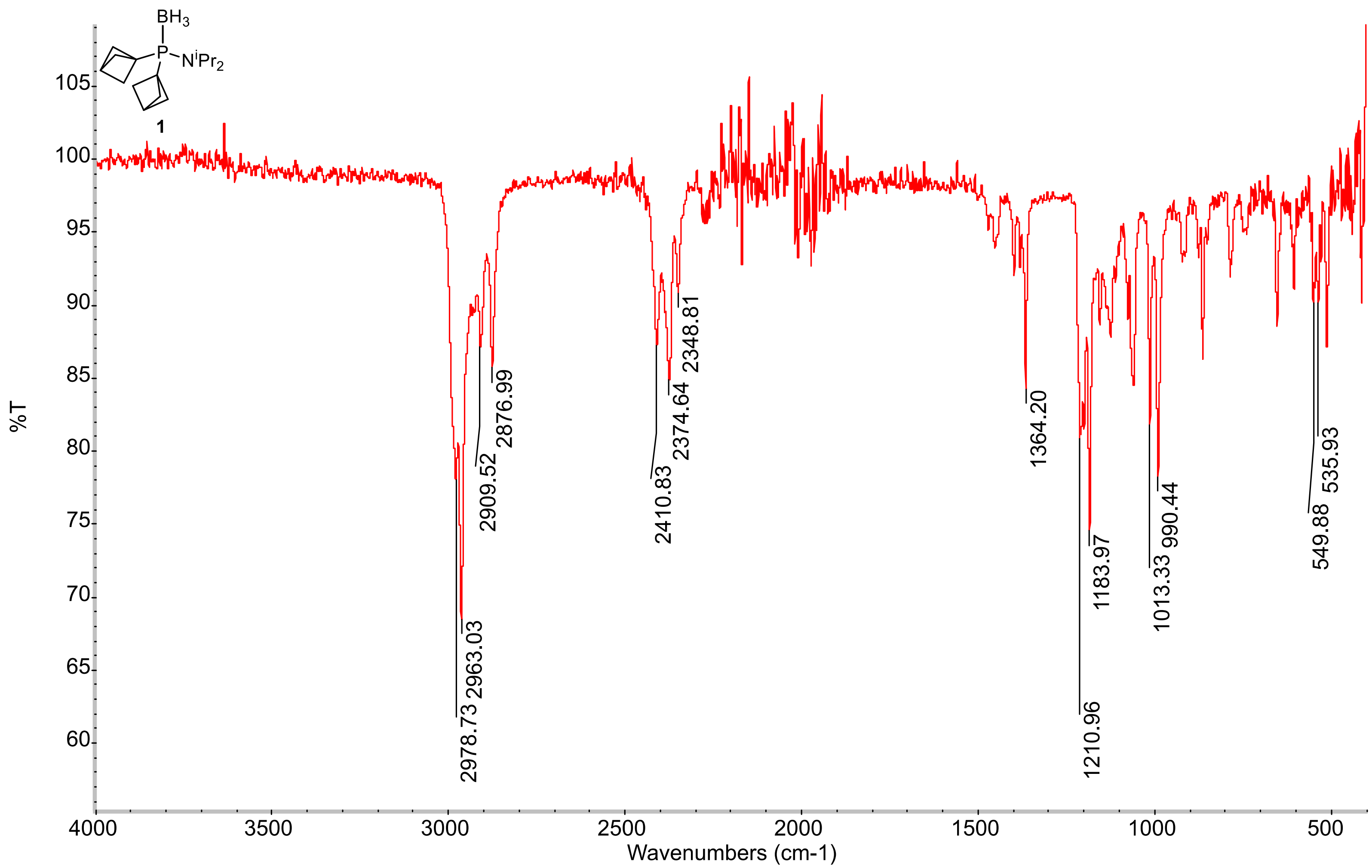

Figure S4. ATR-IR Spectrum of $Bcp_2PN^iPr_2 \cdot BH_3$ (**1**), thin film.

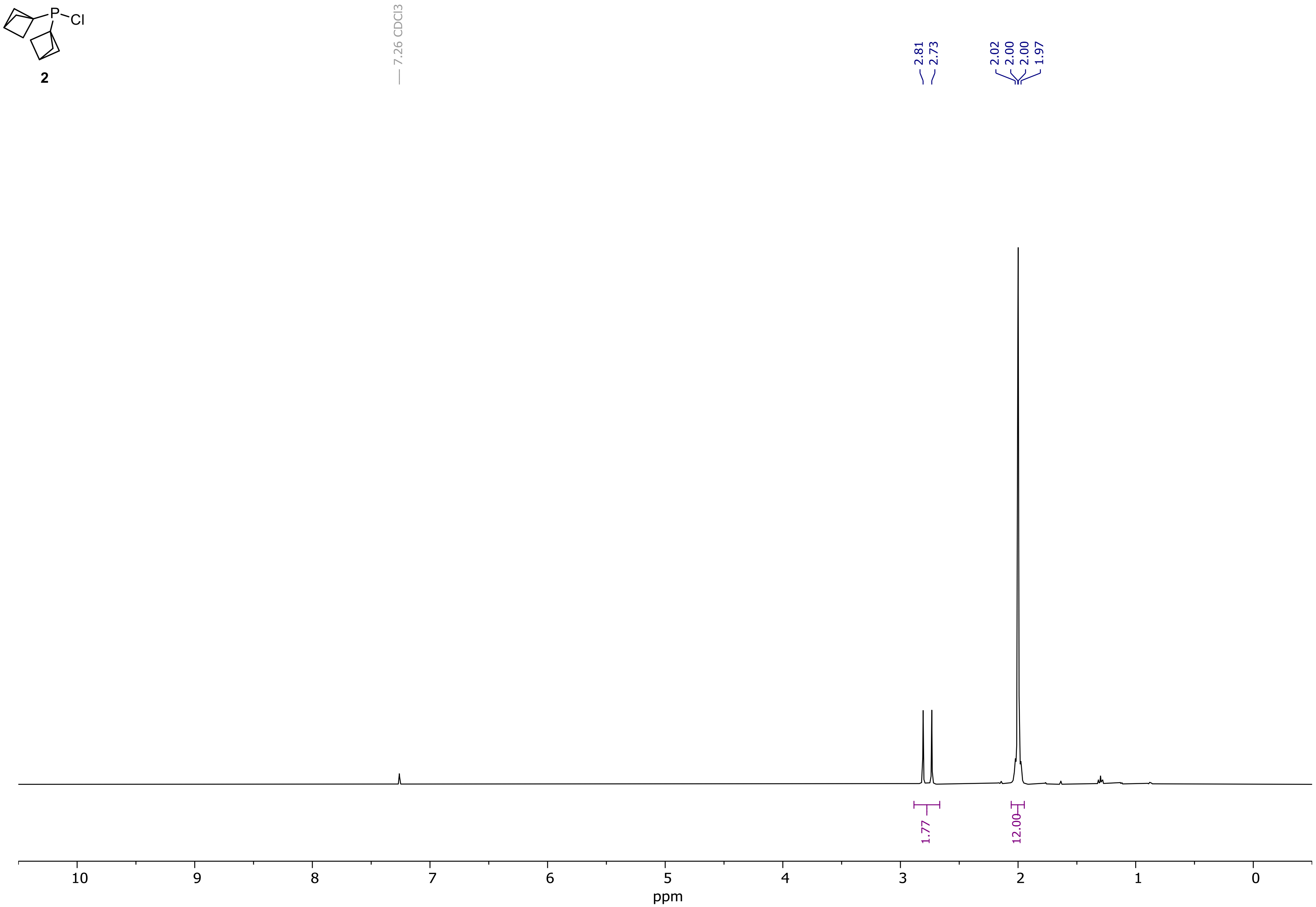

Figure S5. ^1H NMR Spectrum of Bcp_2PCl (**2**) (400 MHz, $CDCl_3$).

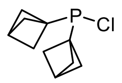

2

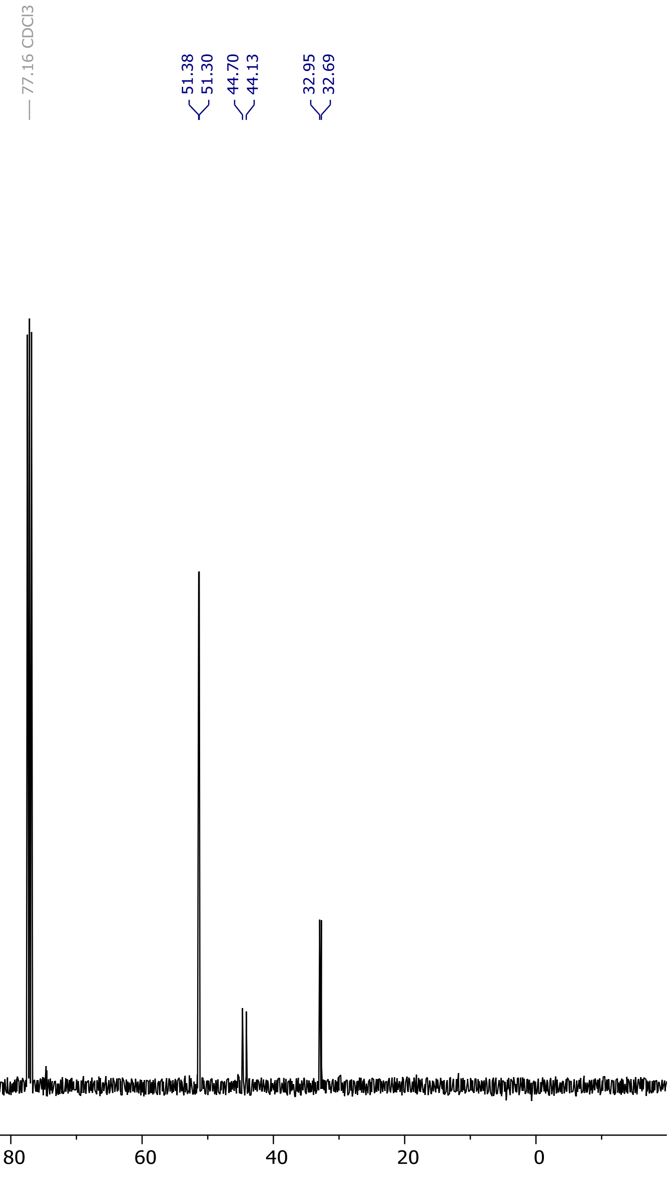

Figure S6. $^{13}C\{^1H\}$ NMR Spectrum of Bcp_2PCl (**2**) (101 MHz, $CDCl_3$).

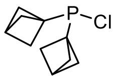

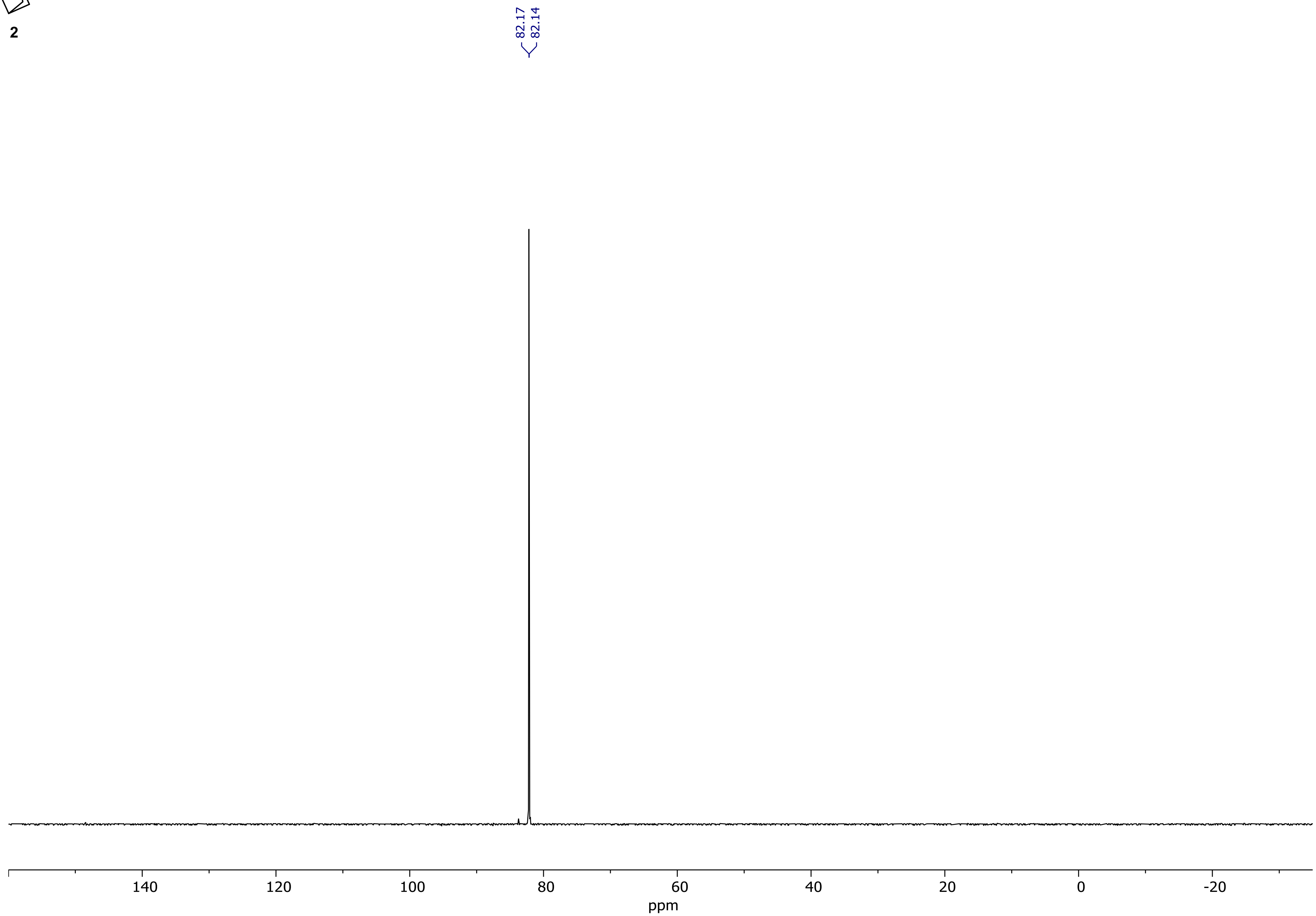

Figure S7. $^{31}P\{^{1}H\}$ NMR Spectrum of Bcp_2PCl (**2**) (162 MHz, $CDCl_3$).

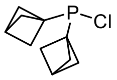

2

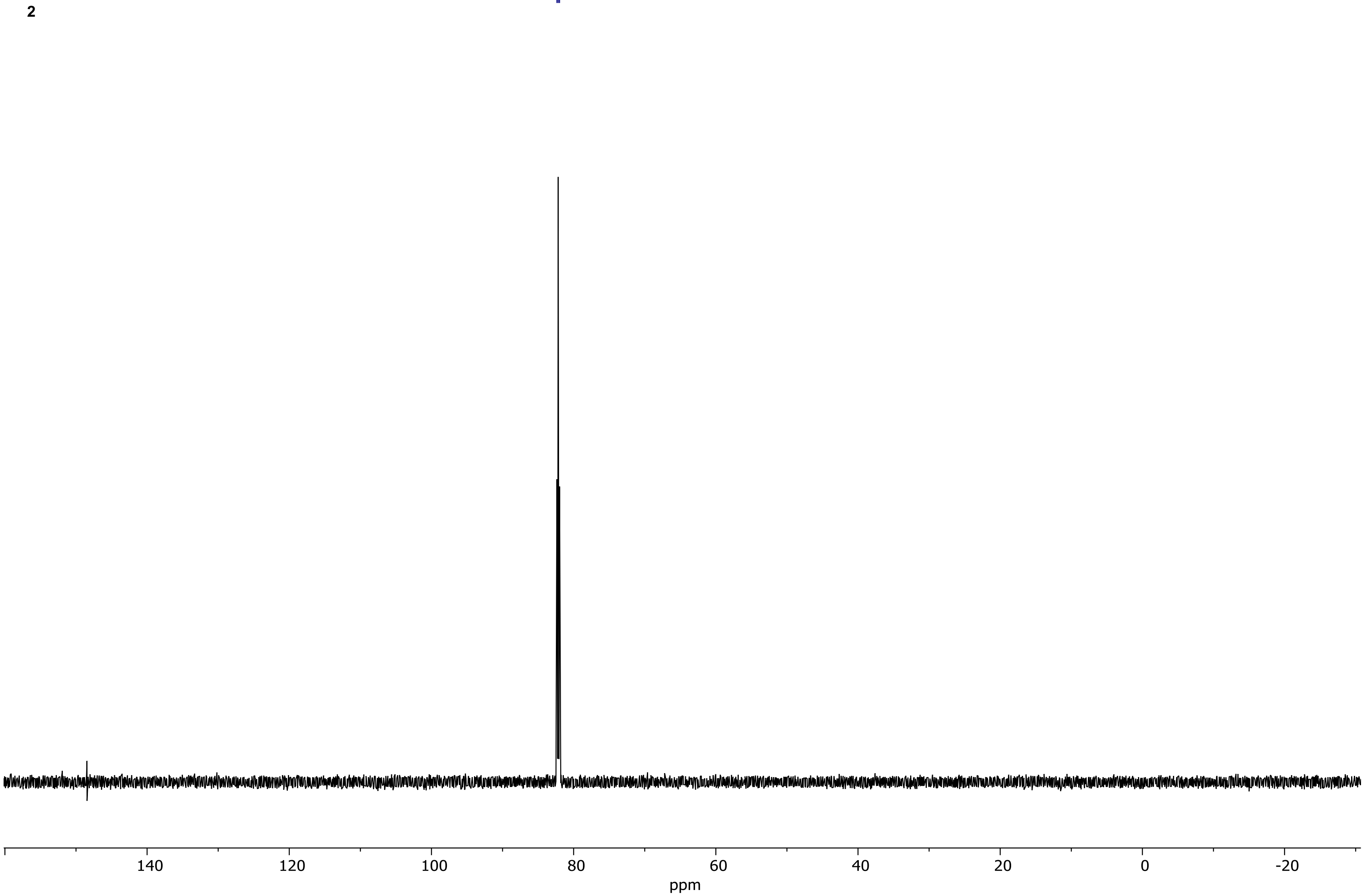

Figure S8. ^{31}P NMR Spectrum of Bcp_2PCl (**2**) (162 MHz, $CDCl_3$).

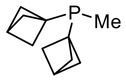

3

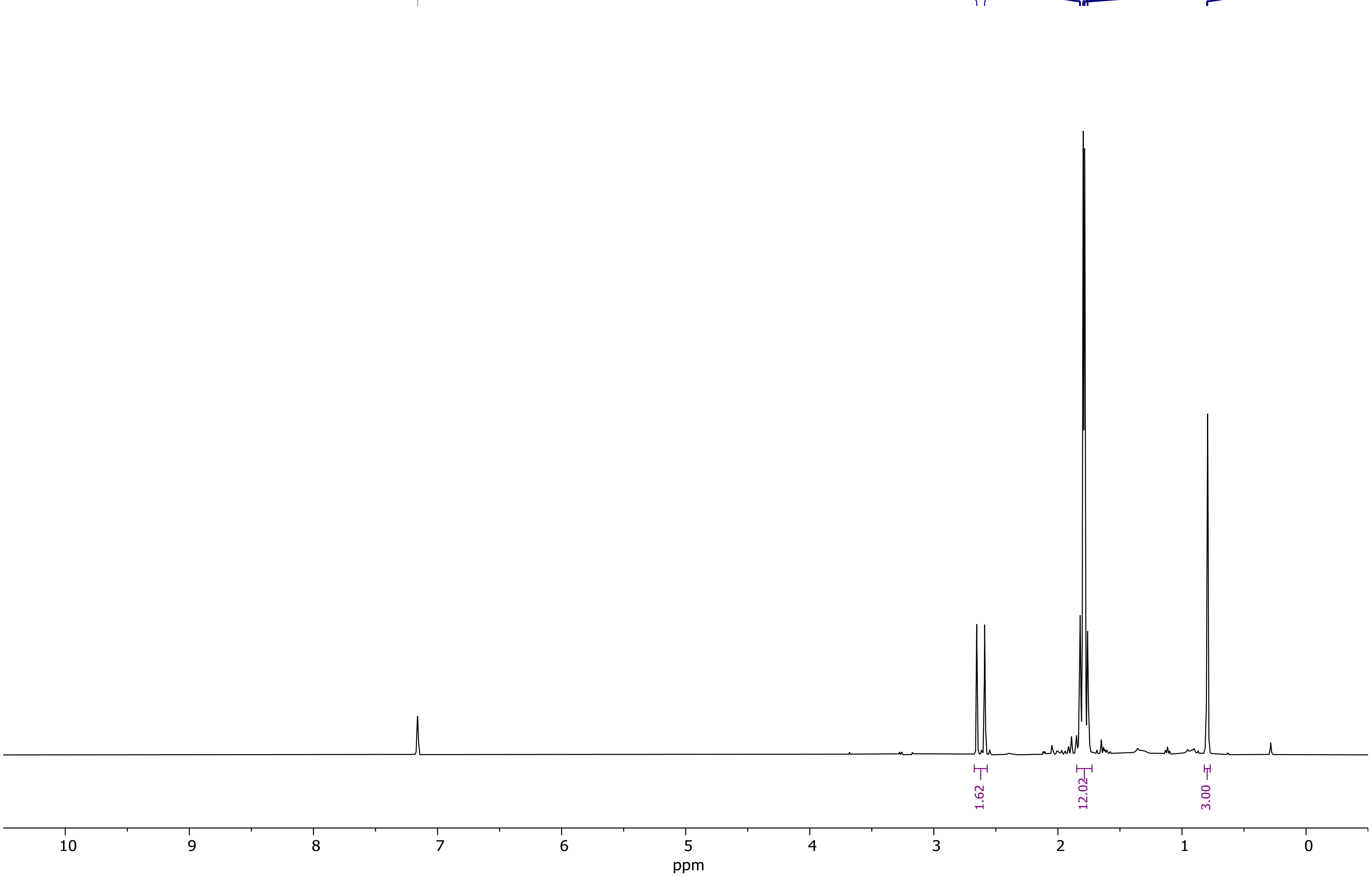

Figure S9. ^{1}H NMR Spectrum of Bcp_2PMe (**3**) (400 MHz, C_6D_6).

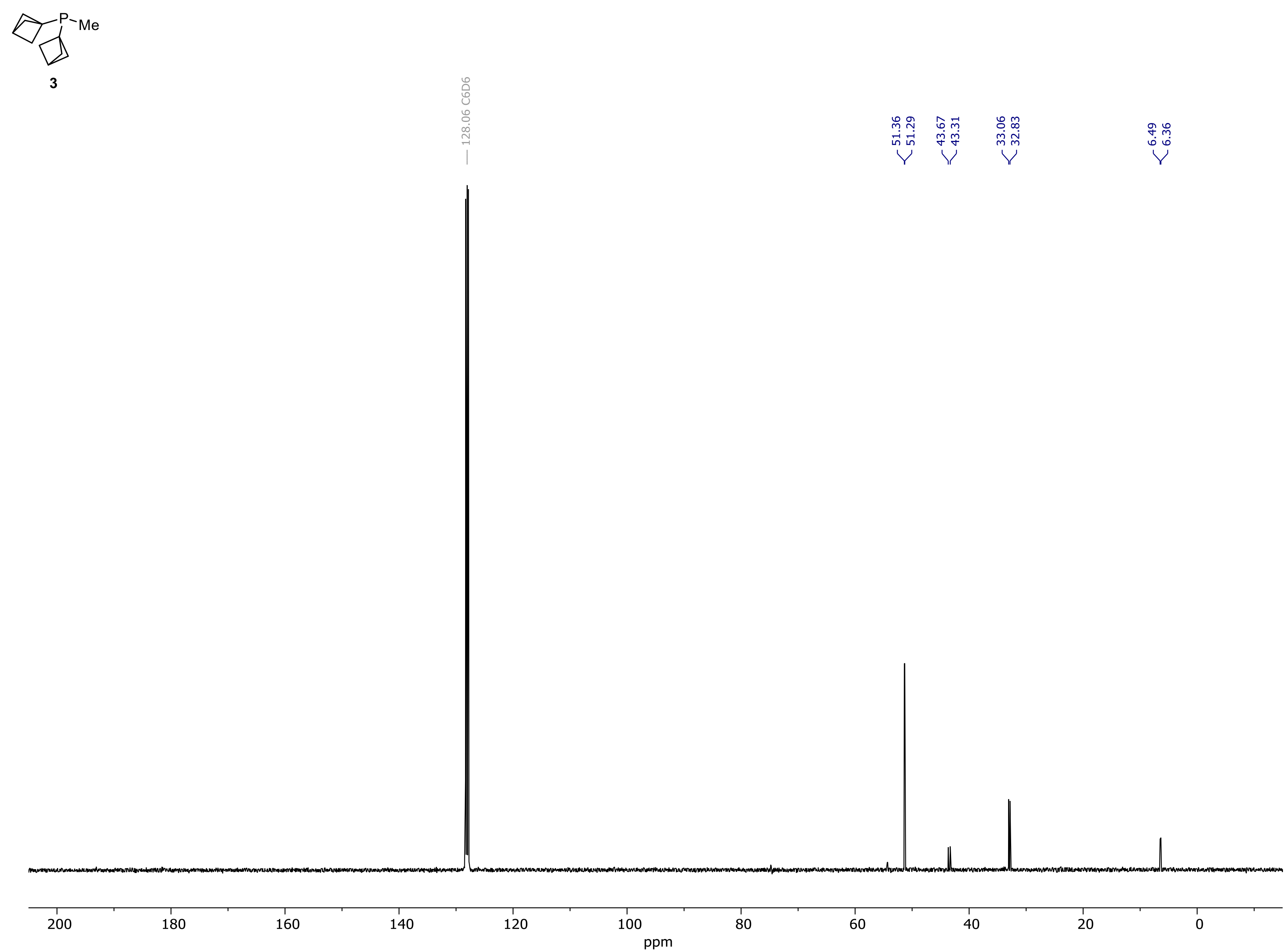

Figure S10. $^{13}C\{^1H\}$ NMR Spectrum of Bcp_2PMe (**3**) (101 MHz, C_6D_6).

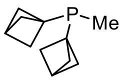

3

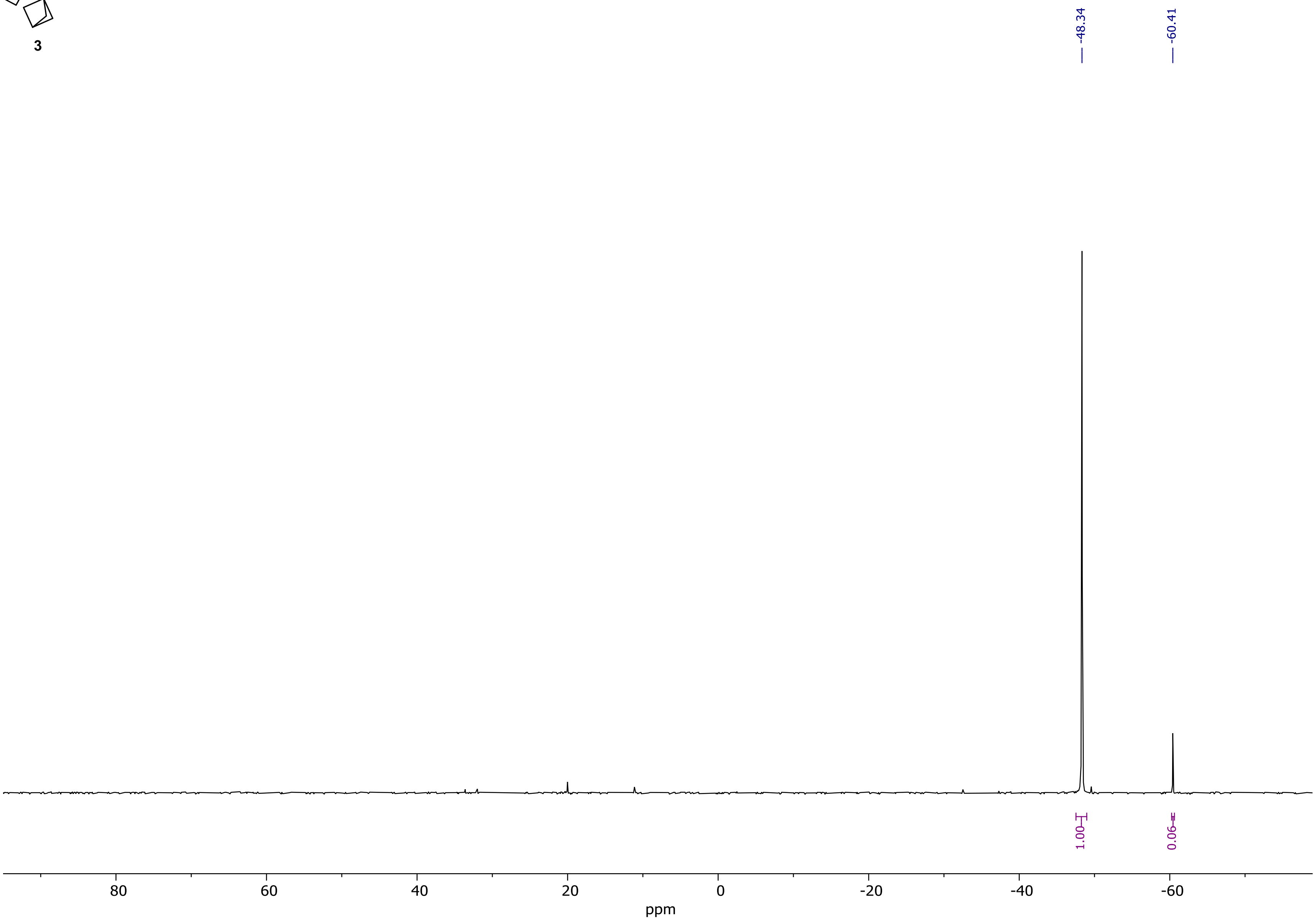

Figure S11. $^{31}P\{^{1}H\}$ NMR Spectrum of Bcp_2PMe (**3**) (162 MHz, C_6D_6).

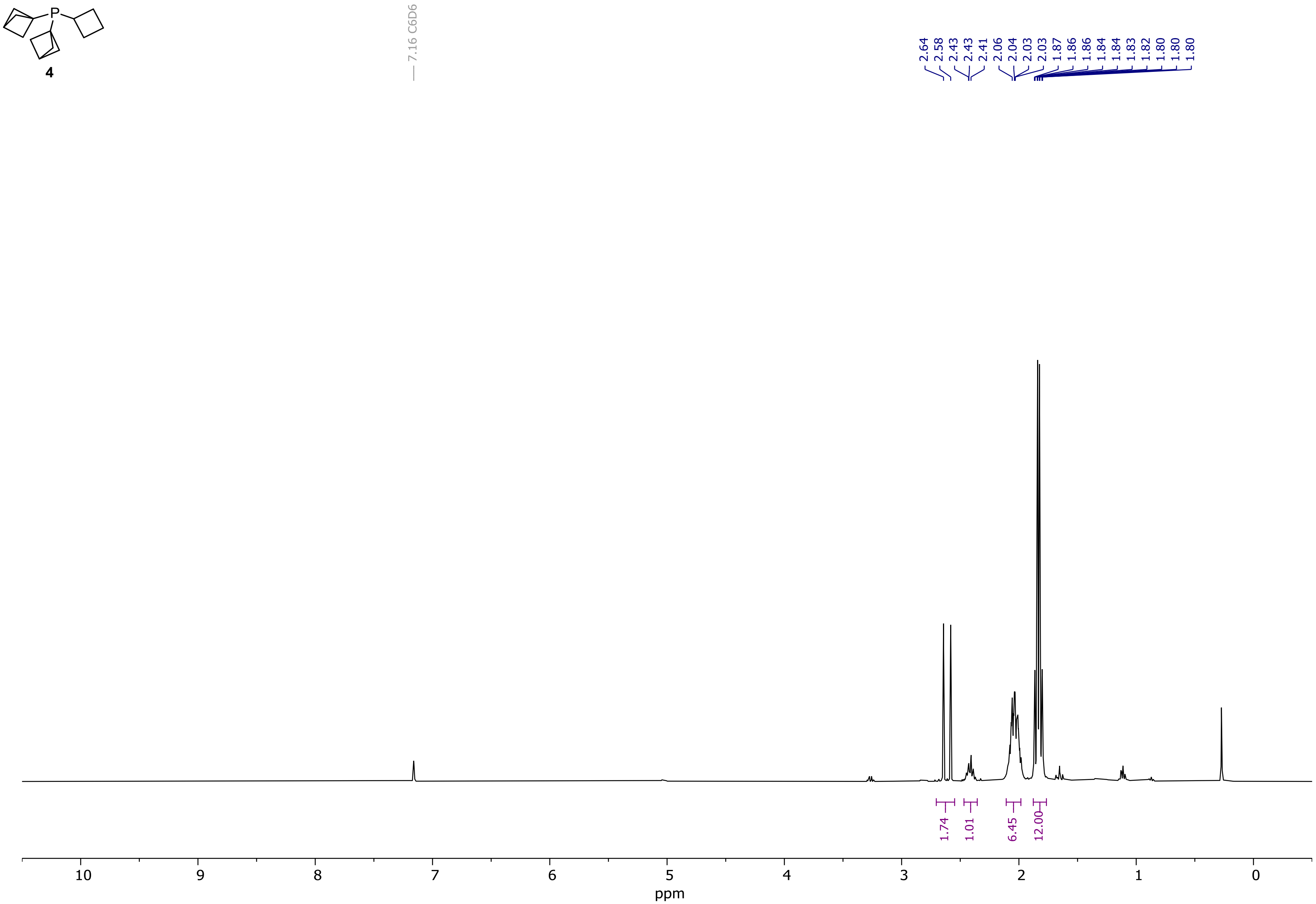

Figure S12. ^{1}H NMR Spectrum of Bcp_2PCyb (**4**) (400 MHz, C_6D_6).

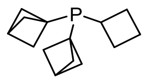

4

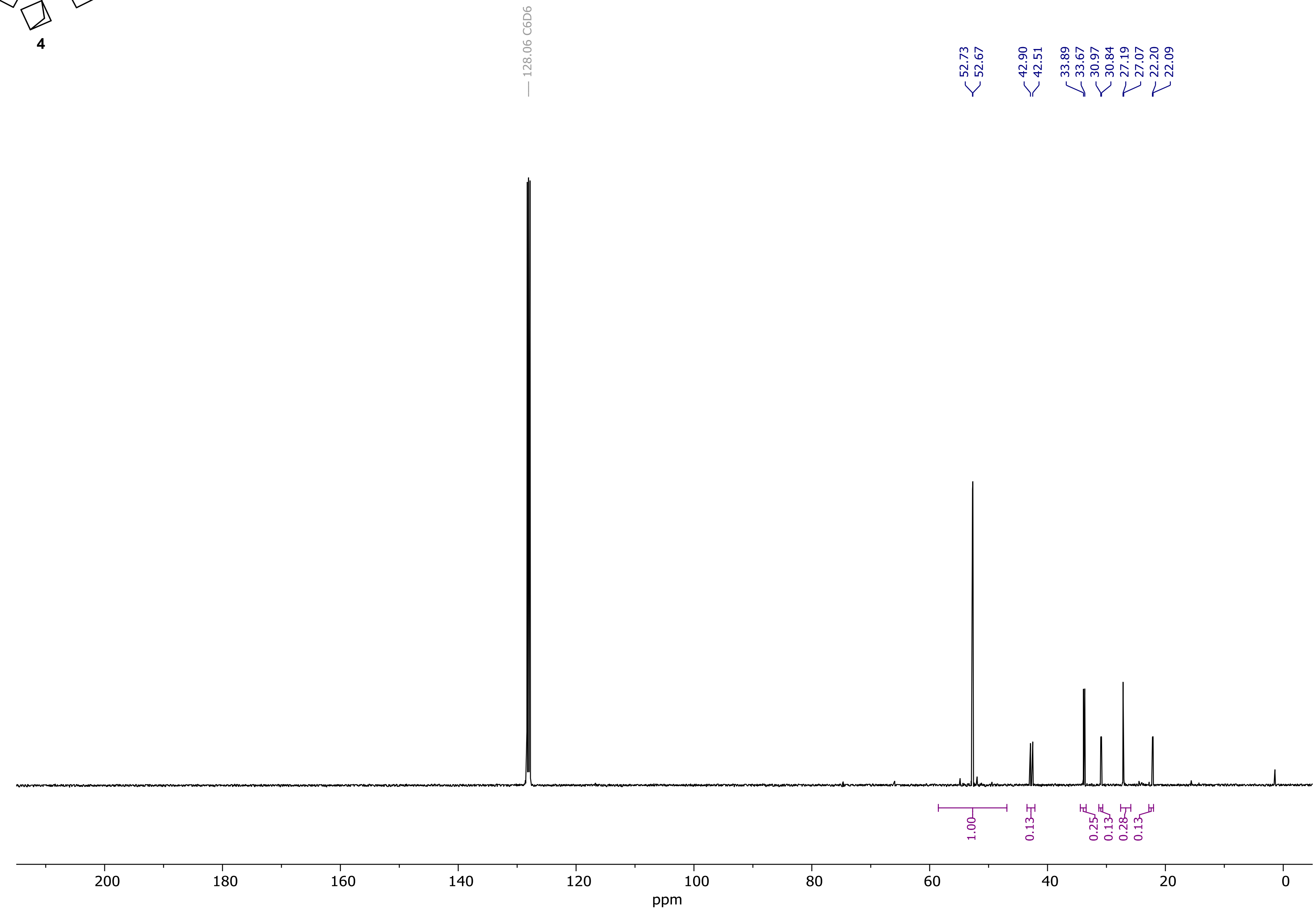

Figure S13. $^{13}C\{^{1}H\}$ NMR Spectrum of Bcp_2PCyb (**4**) (101 MHz, C_6D_6).

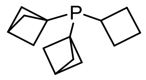

4

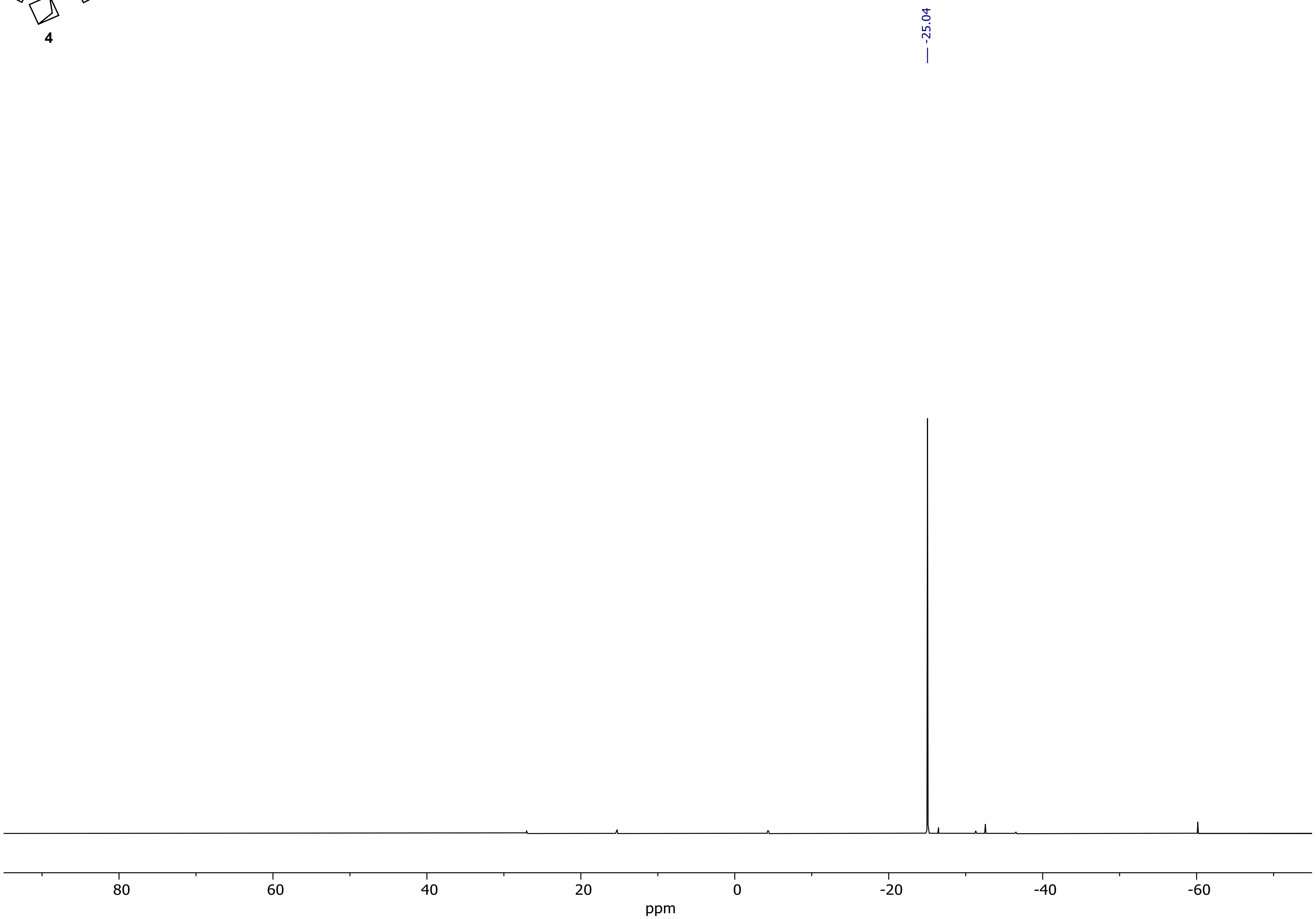

Figure S14. $^{31}P\{^1H\}$ NMR Spectrum of Bcp_2PCyb (**4**) (162 MHz, C_6D_6).

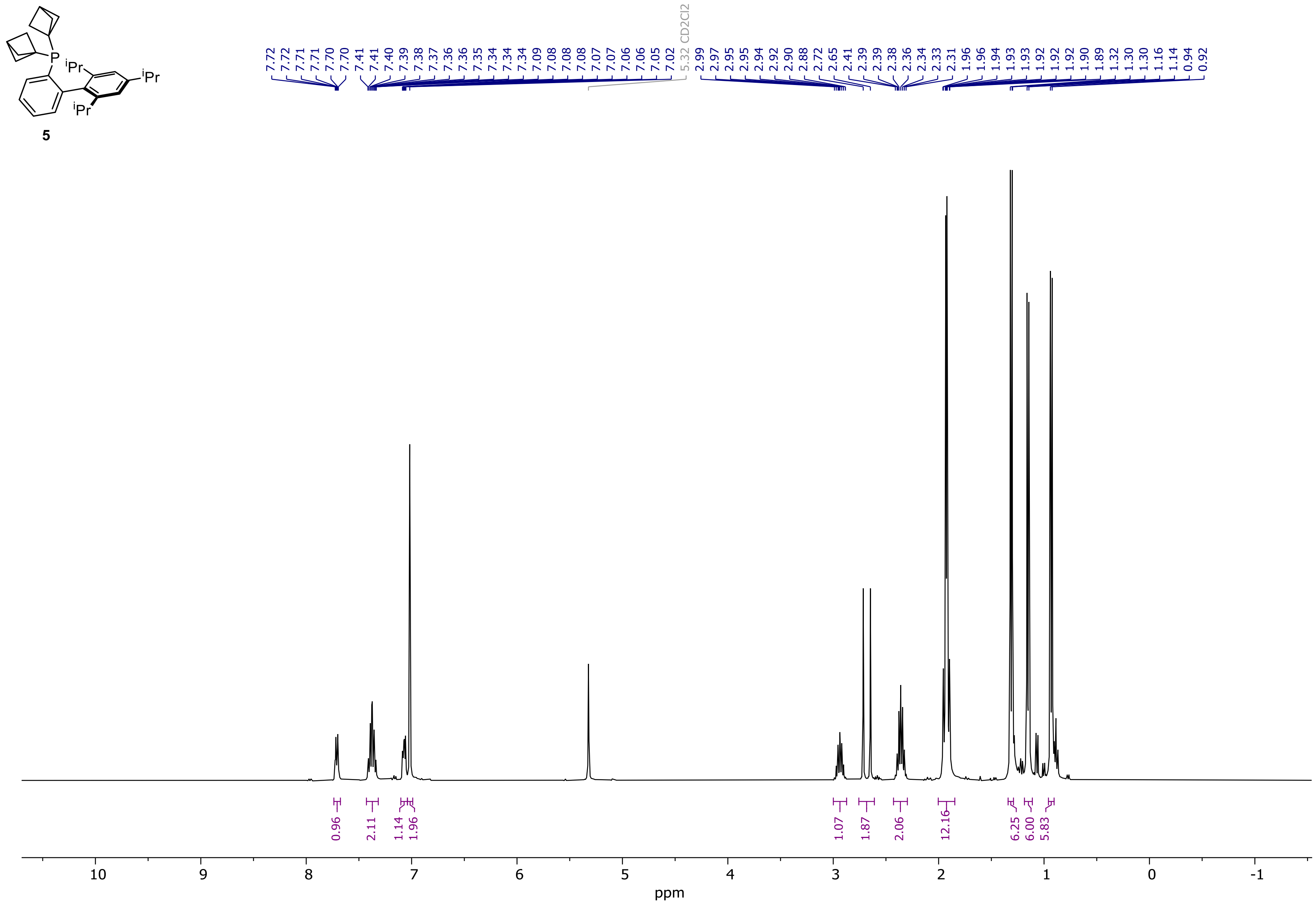

Figure S15. ^{1}H NMR Spectrum of BcpXPhos (**5**) (400 MHz, CD_2Cl_2).

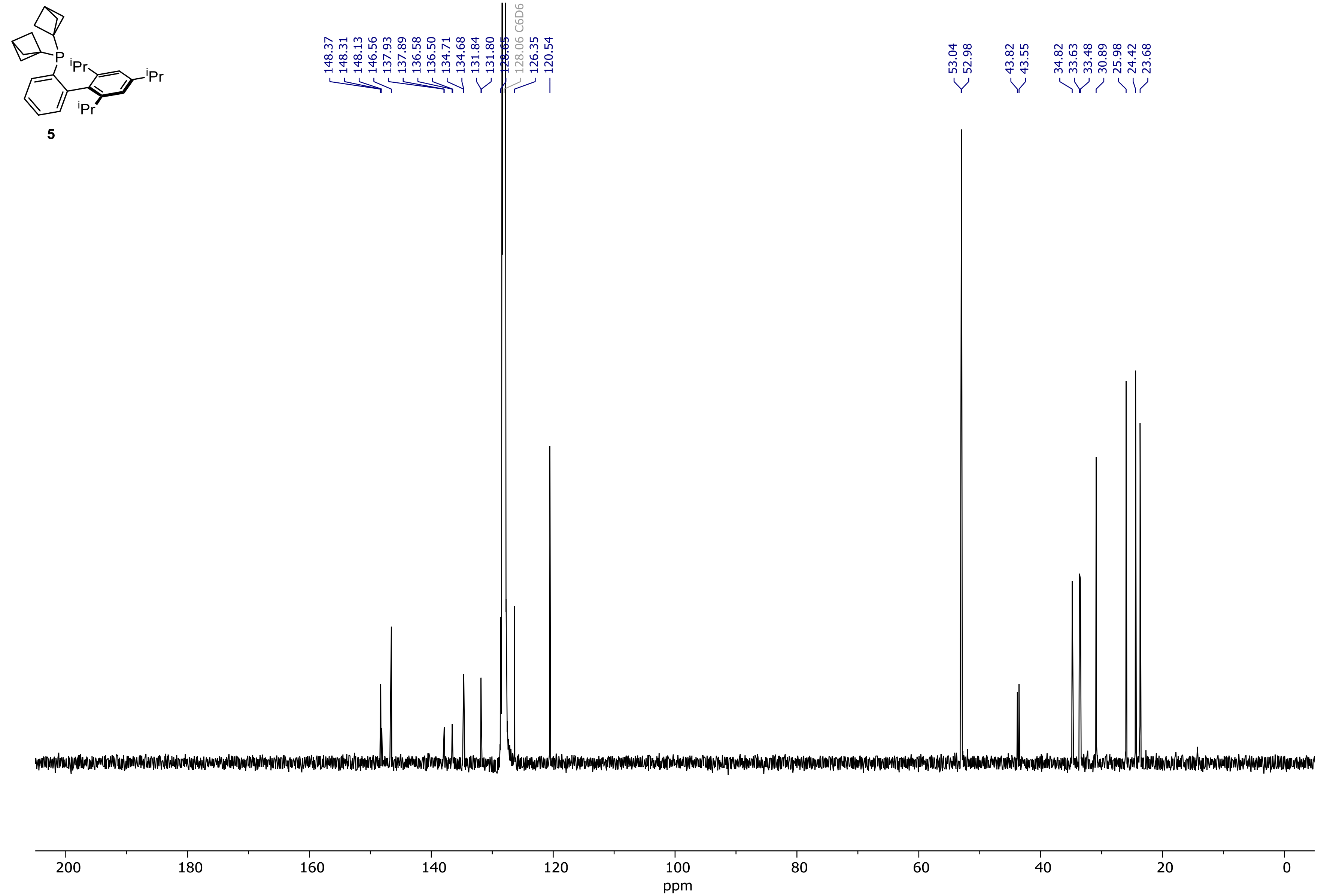

Figure S16. $^{13}C\{^{1}H\}$ NMR Spectrum of BcpXPhos (**5**) (151 MHz, C_6D_6).

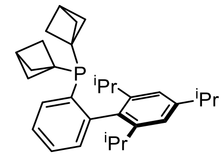

5

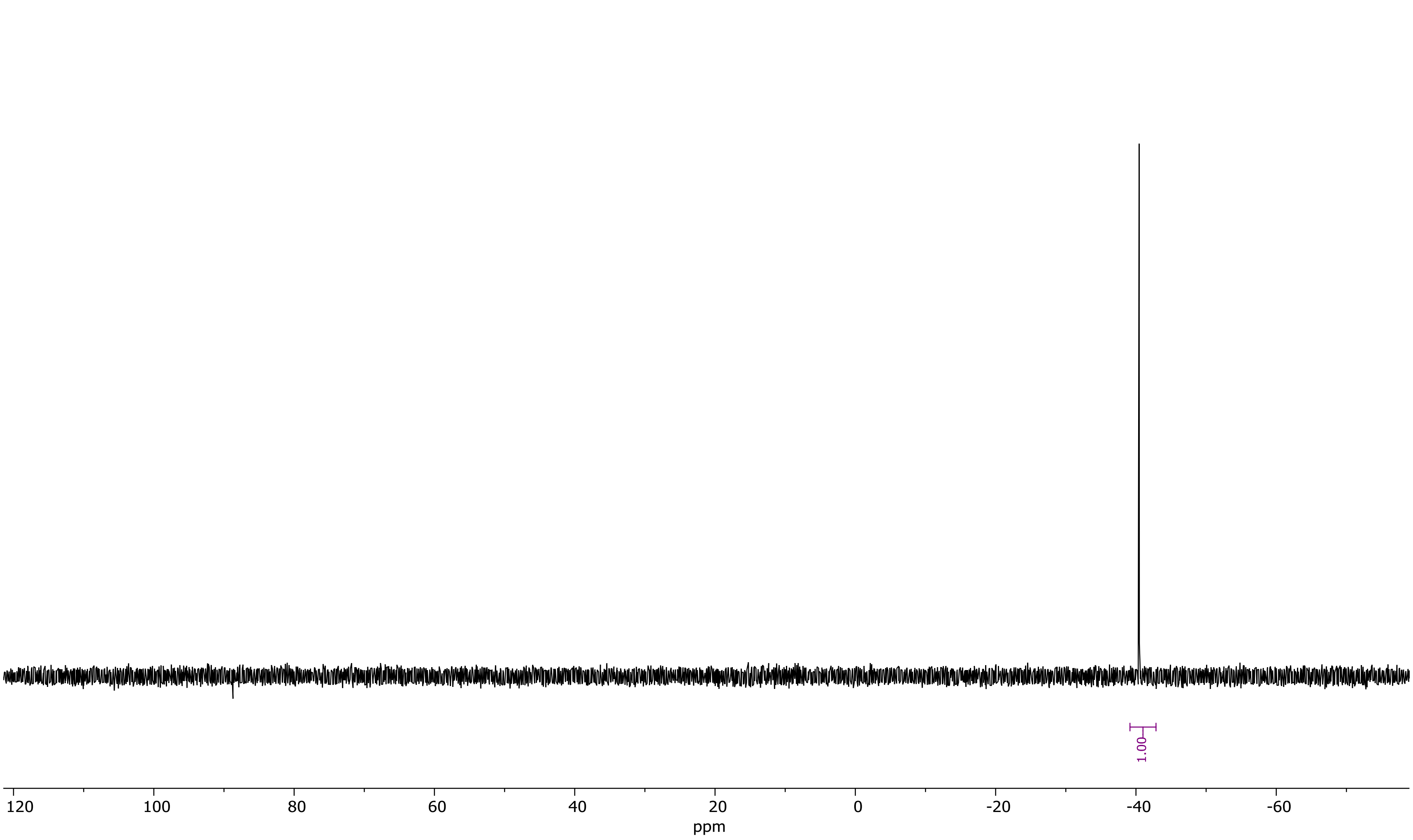

Figure S17. $^{31}P\{^{1}H\}$ NMR Spectrum of BcpXPhos (**5**) (162 MHz, C_6D_6).

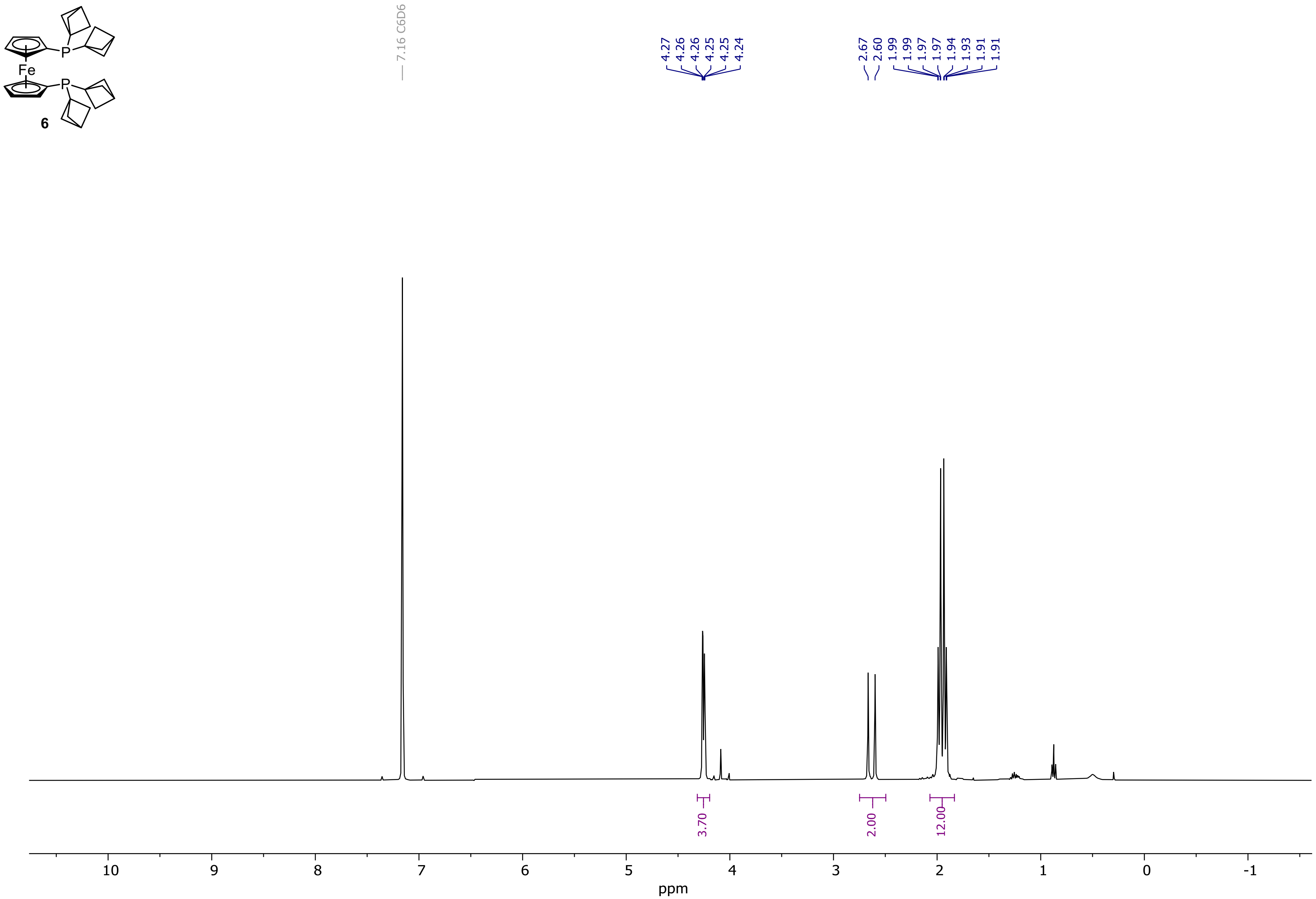

Figure S18. ^{1}H NMR Spectrum of $(Bcp_2P)_2Fc$ (**6**) (400 MHz, C_6D_6).

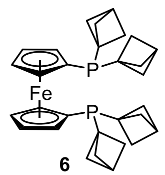

6

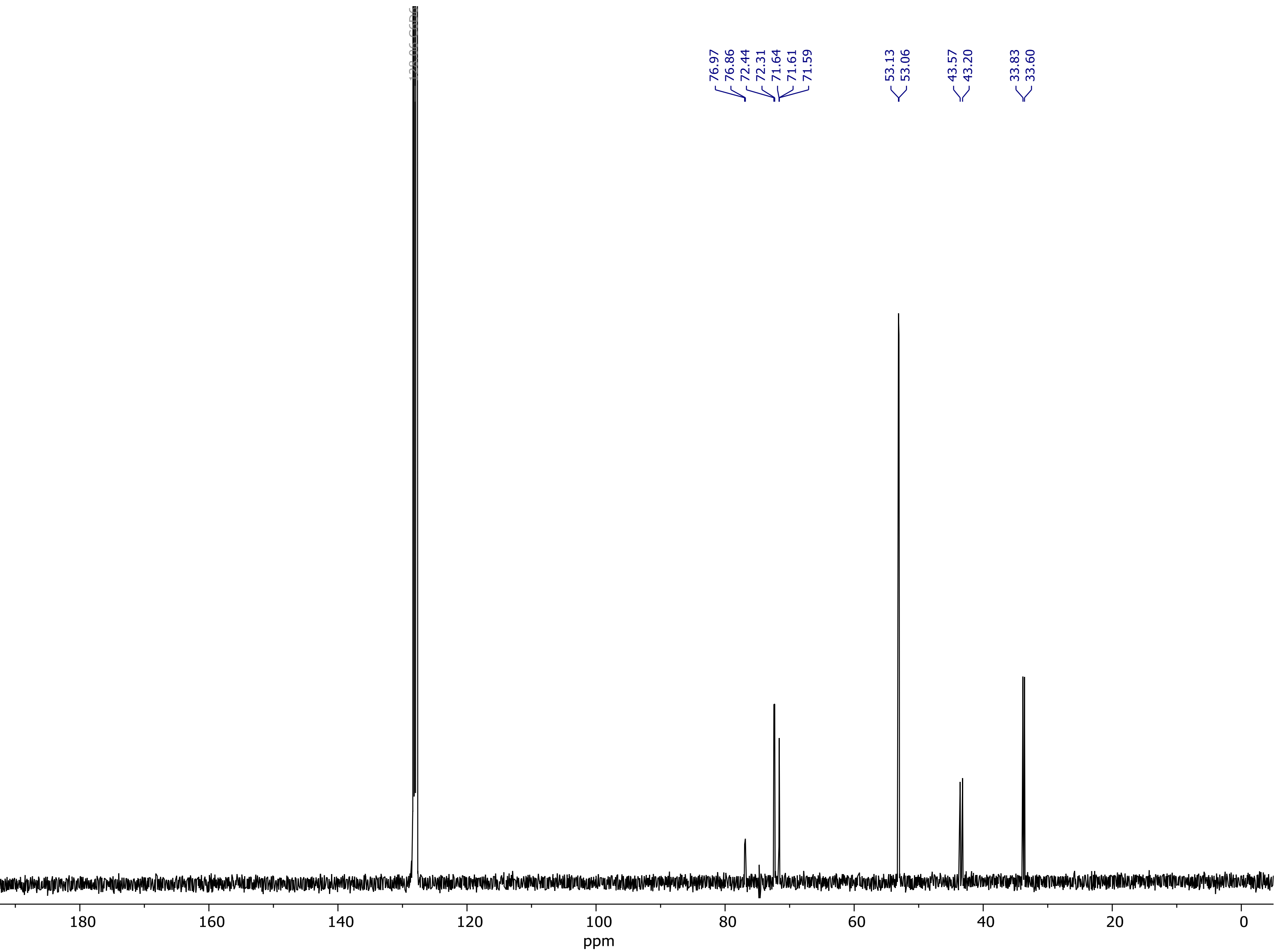

Figure S19. $^{13}C\{^{1}H\}$ NMR Spectrum of $(Bcp_2P)_2Fc$ (**6**) (101 MHz, C_6D_6).

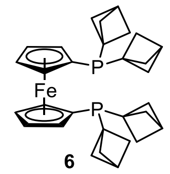

6

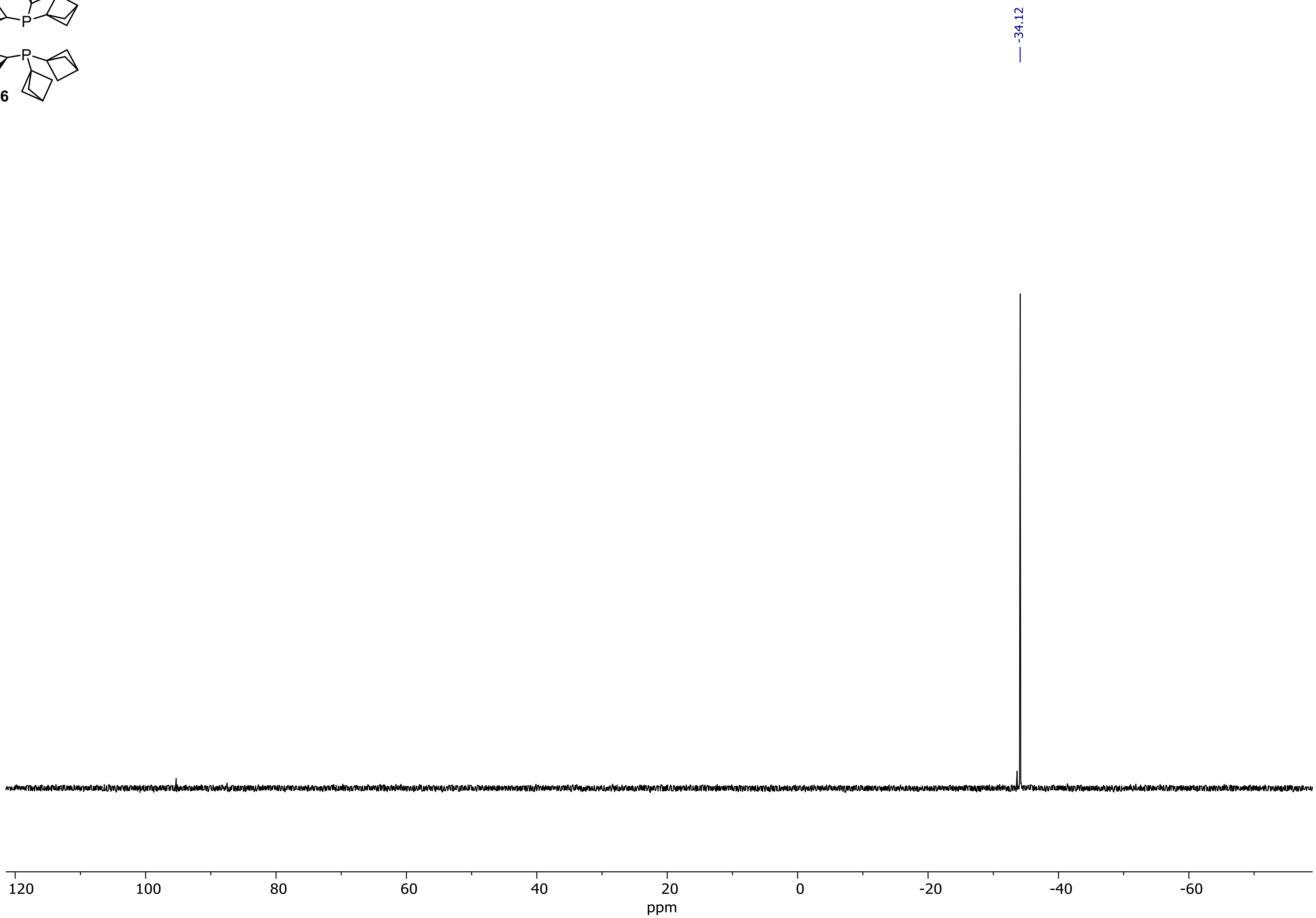

Figure S20. $^{31}P\{^{1}H\}$ NMR Spectrum of $(Bcp_2P)_2Fc$ (**6**) (162 MHz, C_6D_6).

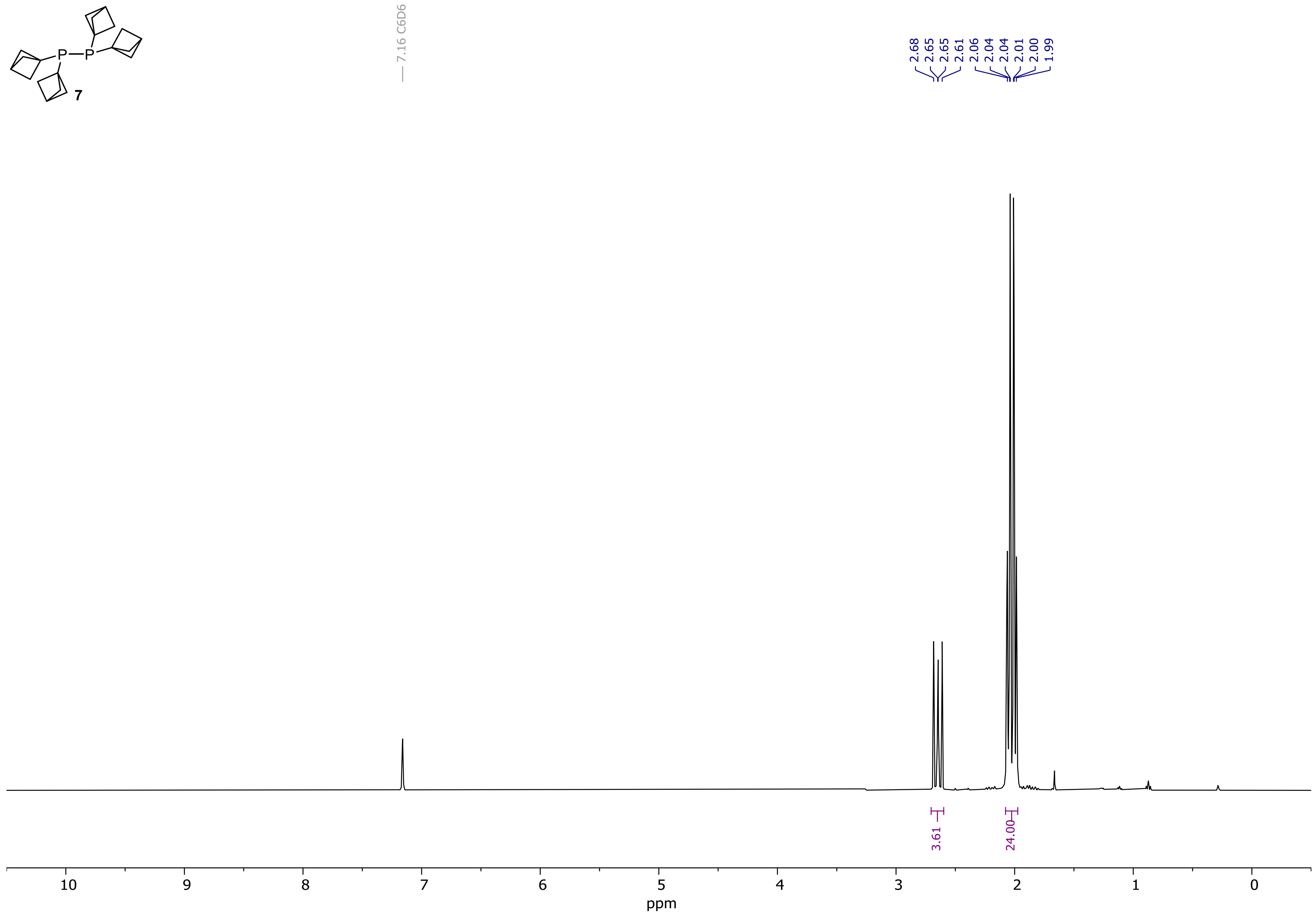

Figure S21. ¹H NMR Spectrum of $(Bcp_2P)_2$ (**7**) (400 MHz, C_6D_6).

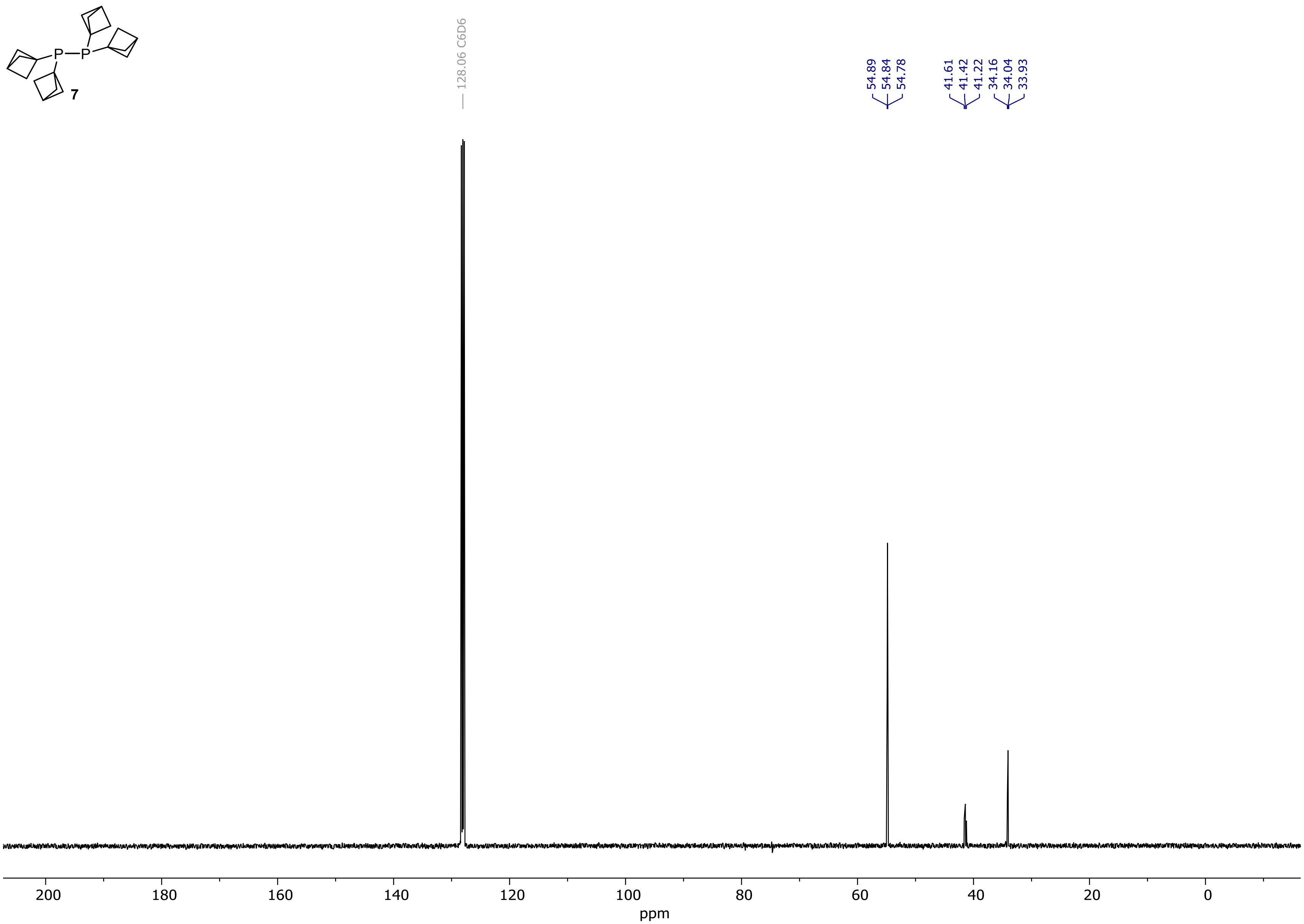

Figure S22. $^{13}C\{^1H\}$ NMR Spectrum of $(Bcp_2P)_2$ (**7**) (101 MHz, C_6D_6).

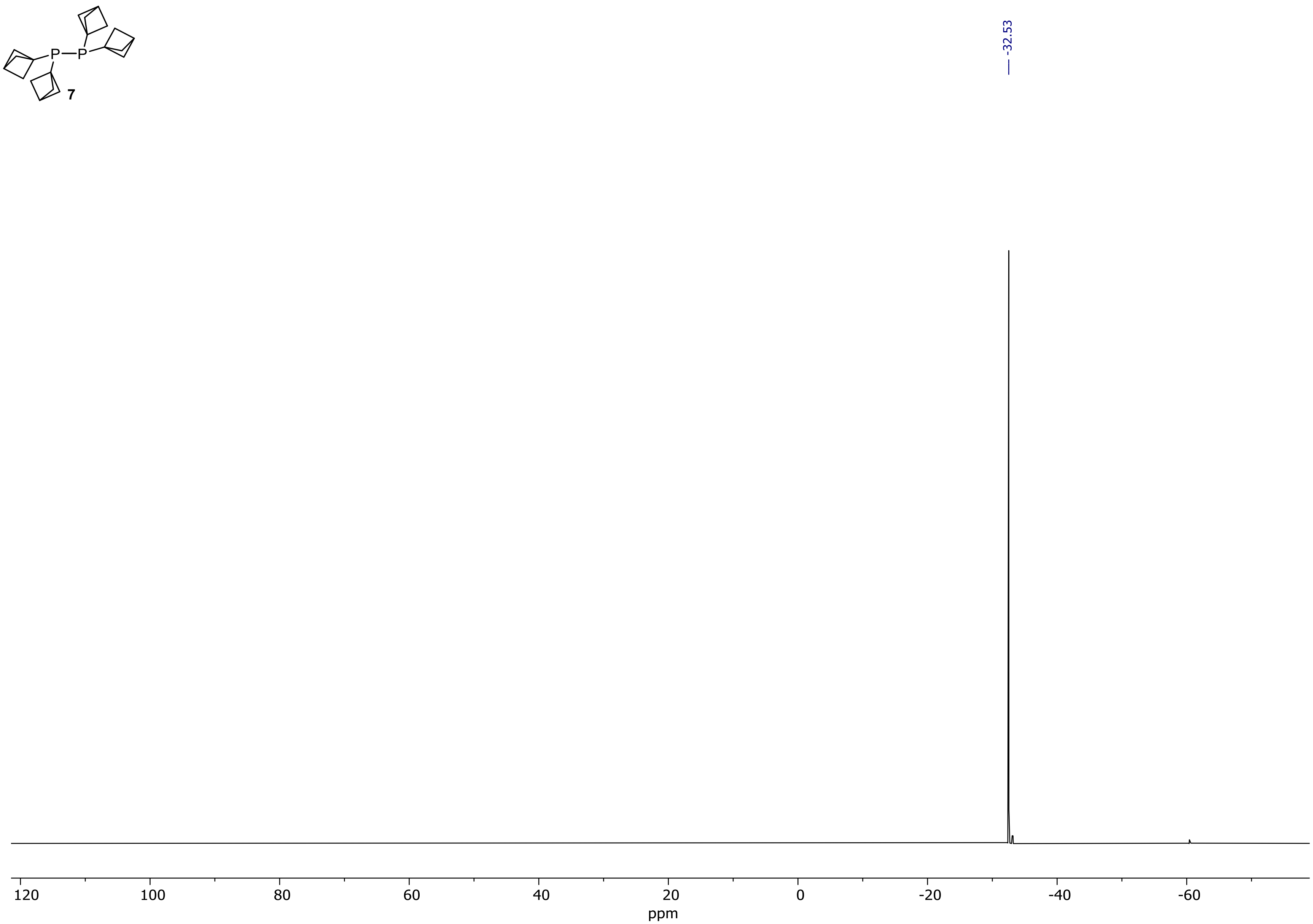

Figure S23. $^{31}P\{^{1}H\}$ NMR Spectrum of $(Bcp_2P)_2$ (**7**) (162 MHz, C_6D_6).

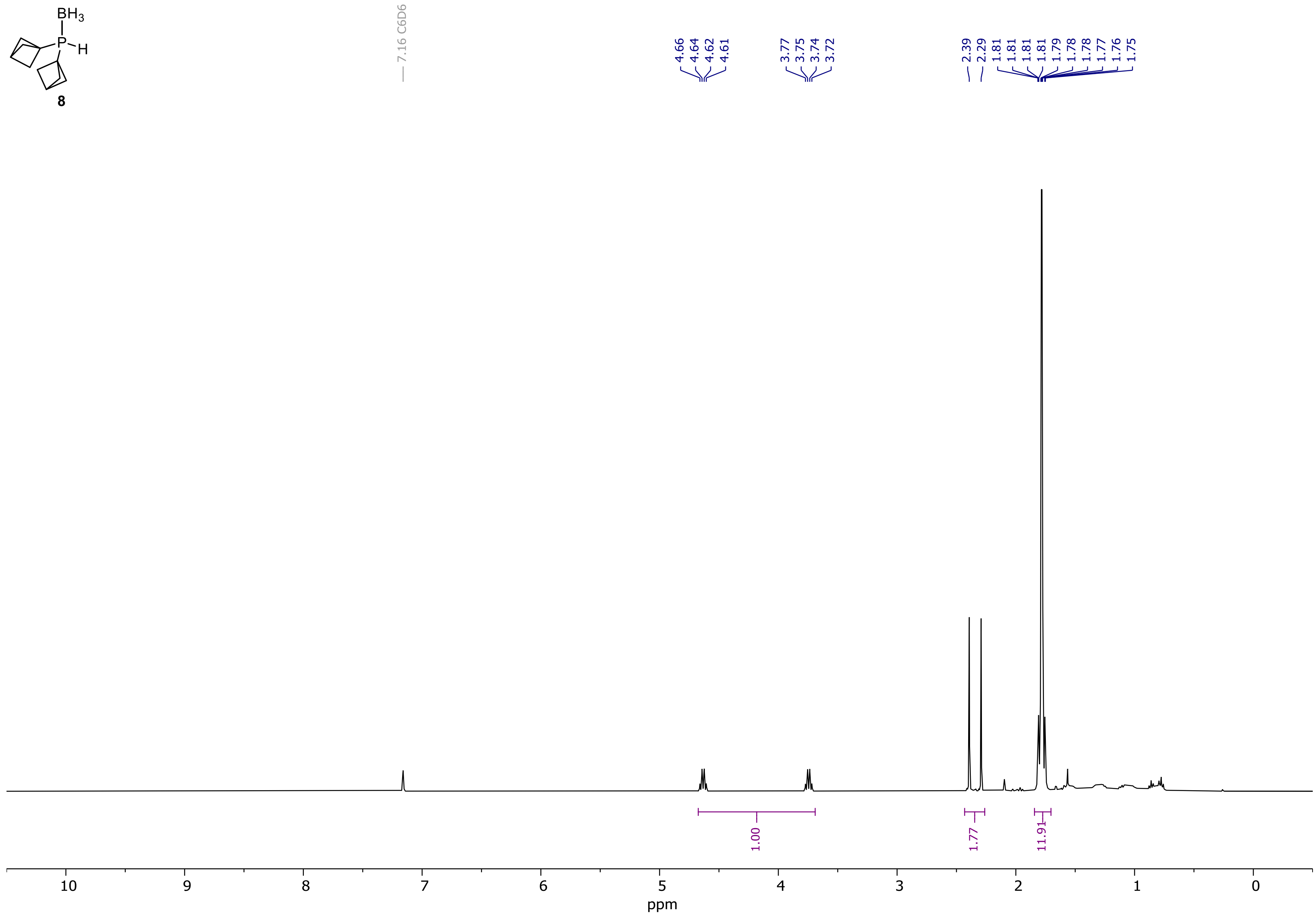

Figure S24. ^{1}H NMR Spectrum of $Bcp_2PH{\cdot}BH_3$ (**8**) (400 MHz, C_6D_6).

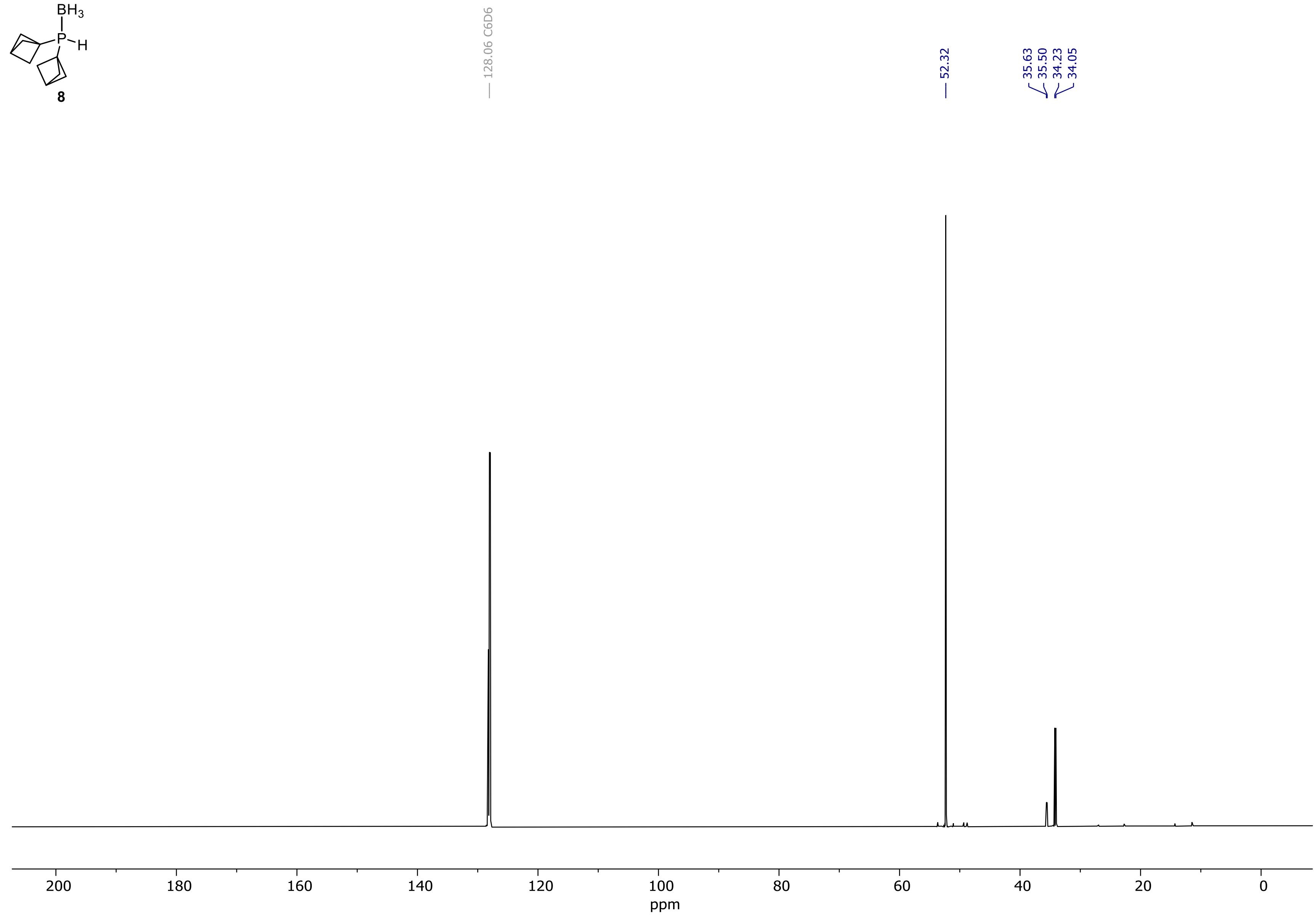

Figure S25. $^{13}C\{^{1}H\}$ NMR Spectrum of $Bcp_2PH{\cdot}BH_3$ (**8**) (151 MHz, C_6D_6).

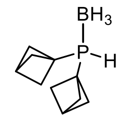

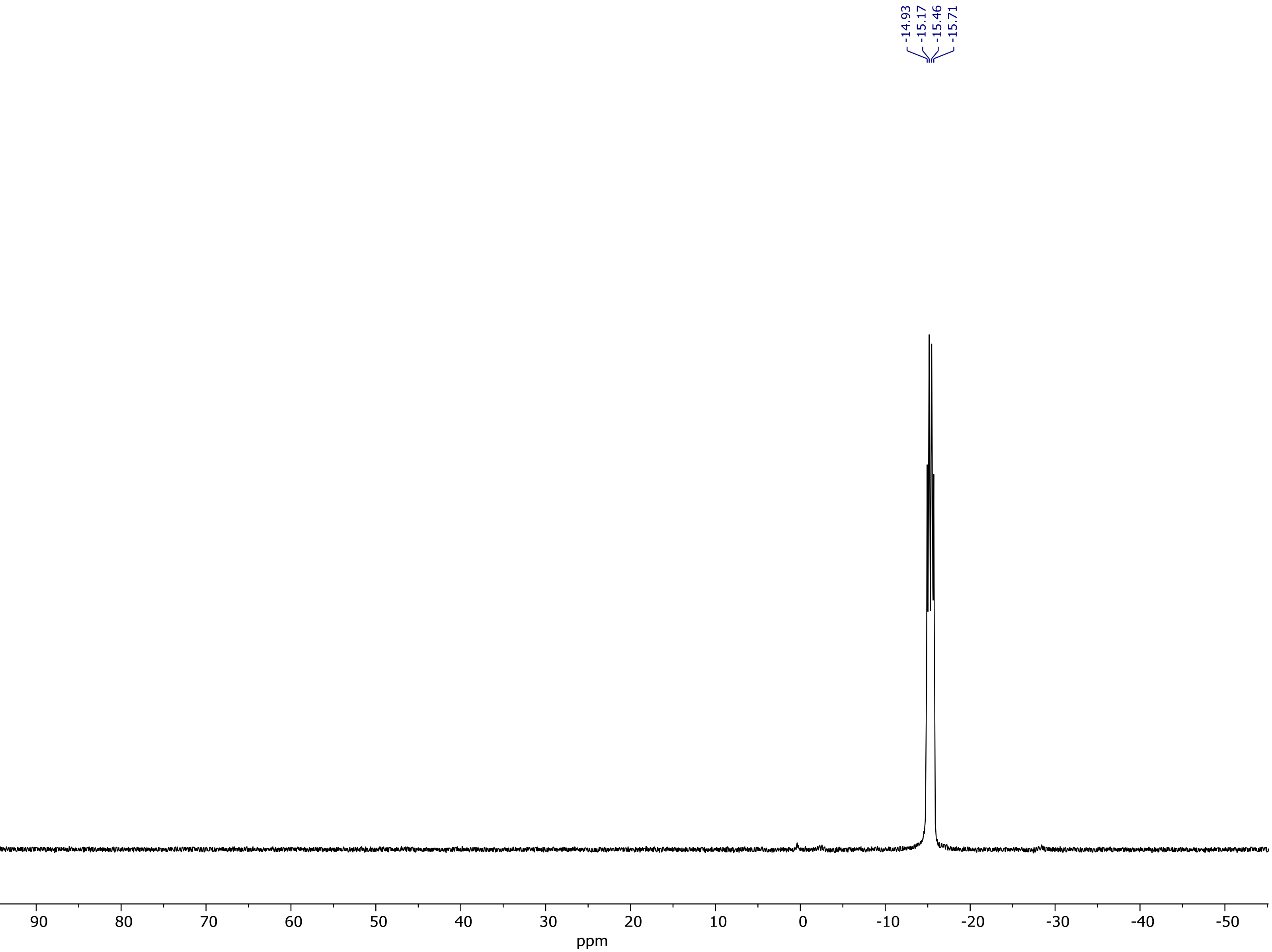

Figure S26. $^{31}P\{^{1}H\}$ NMR Spectrum of $Bcp_2PH{\cdot}BH_3$ (**8**) (162 MHz, C_6D_6).

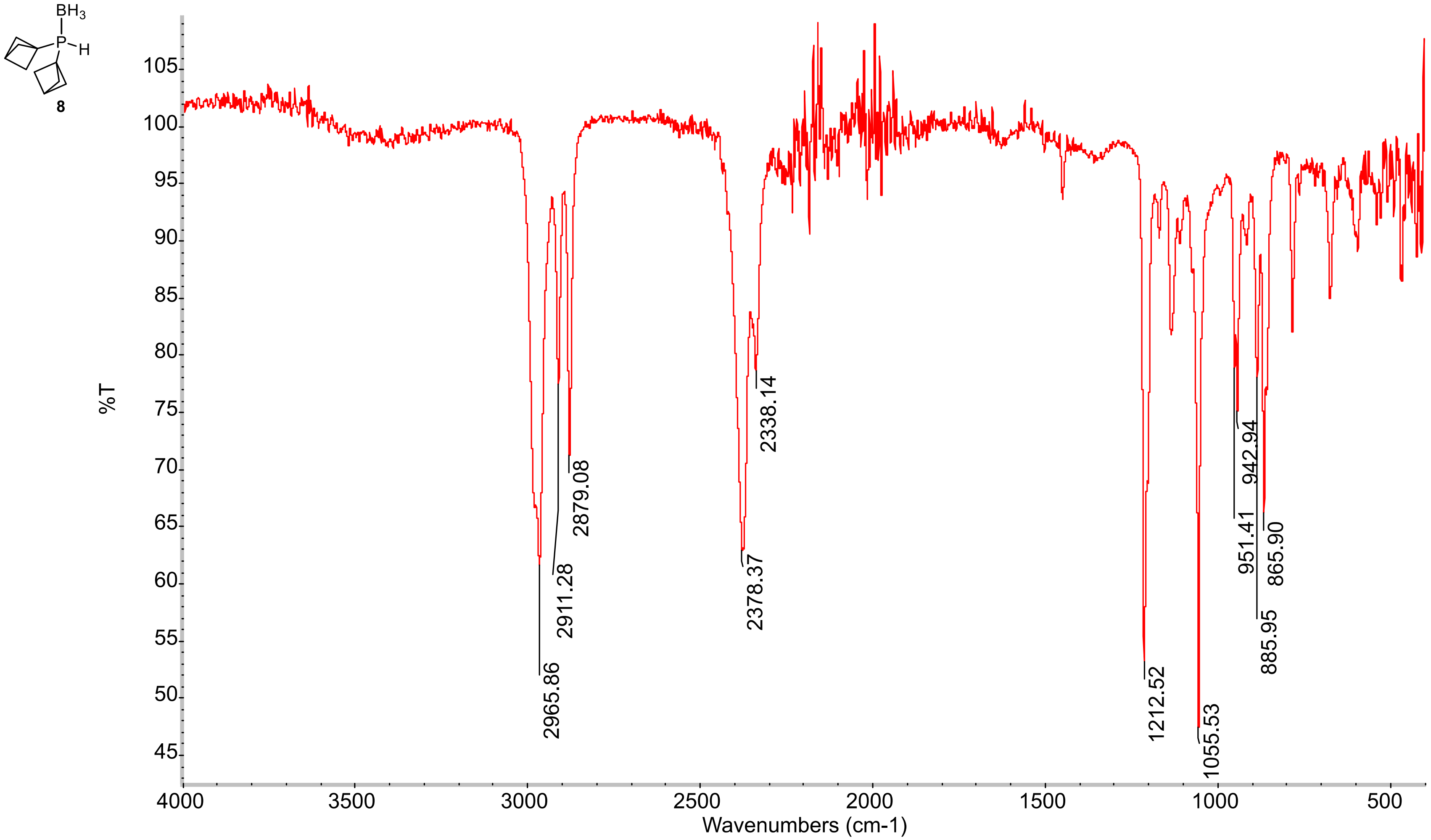

Figure S27. ATR-IR Spectrum of $Bcp_2PH·BH_3$ (**8**), thin film.

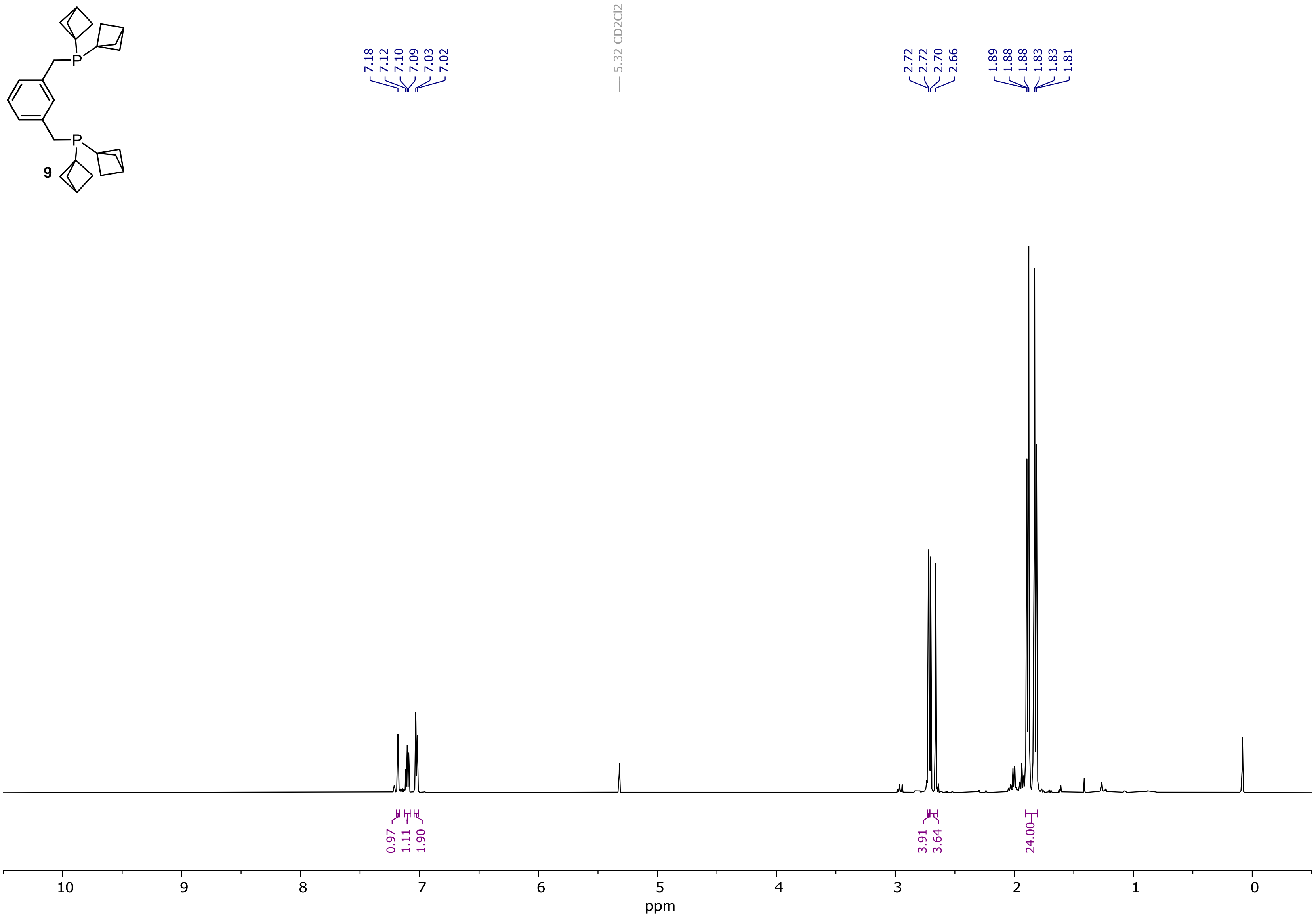

Figure S28. ^{1}H NMR Spectrum of ^{Bcp4}PCP (**9**) (600 MHz, CD_2Cl_2).

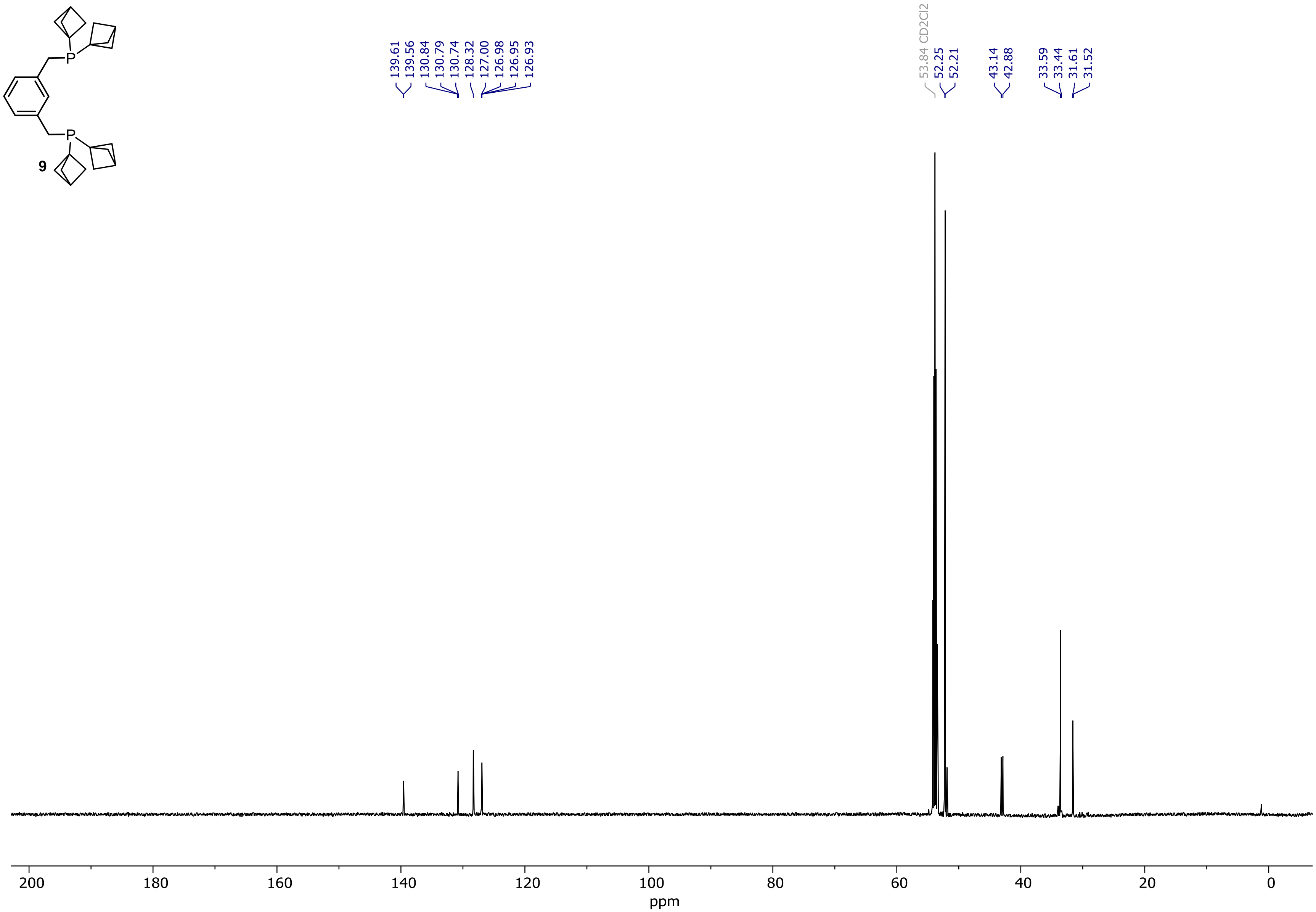

Figure S29. $^{13}C\{^{1}H\}$ NMR Spectrum of ^{Bcp4}PCP (**9**) (151 MHz, CD_2Cl_2).

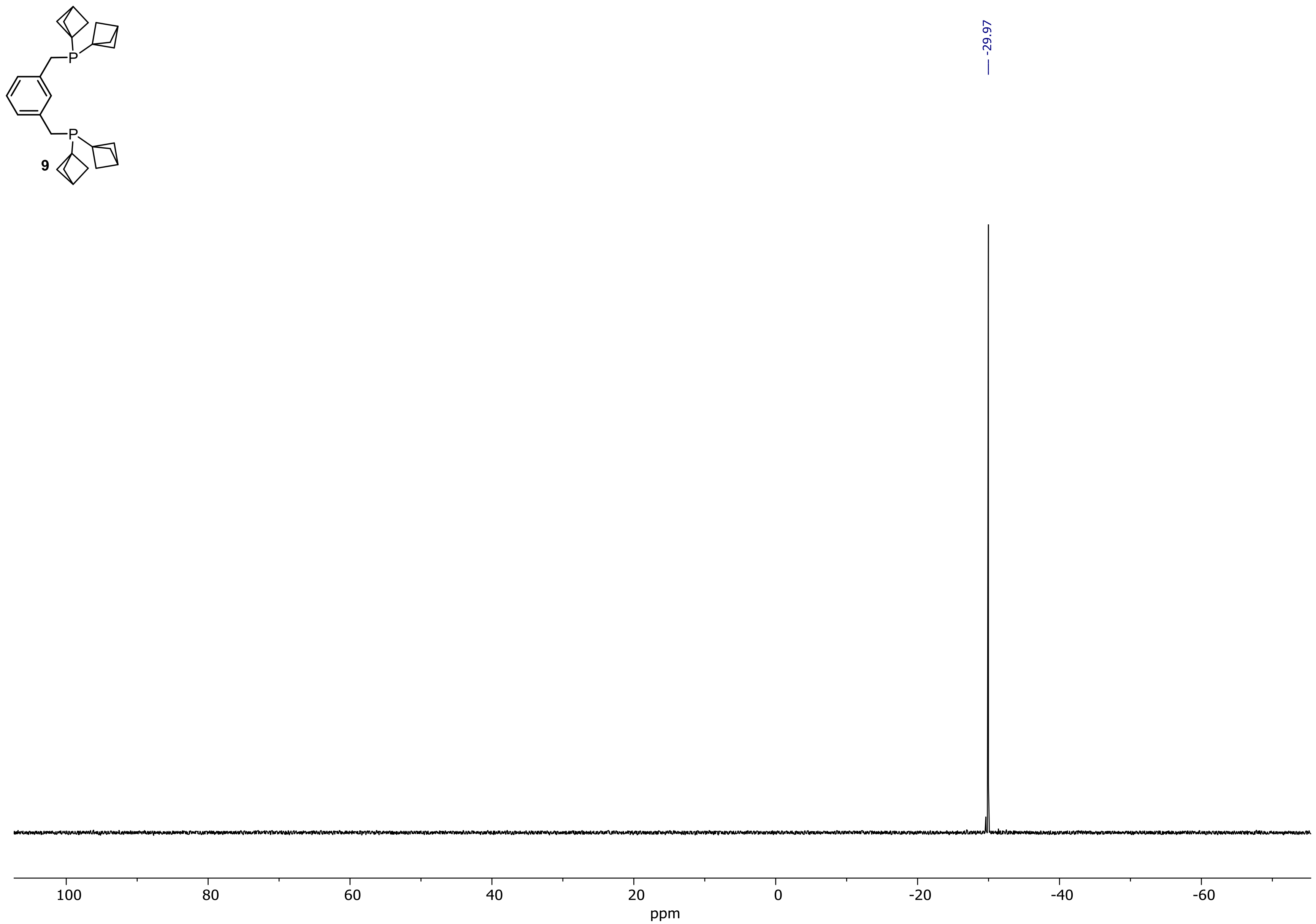

Figure S30. $^{31}P\{^{1}H\}$ NMR Spectrum of ^{Bcp4}PCP (**9**) (162 MHz, C_6D_6).

V. X-ray Crystallographic Data

Details of crystallographic refinement

General Methods. A suitable crystal of each sample was selected for analysis and mounted in a polyimide loop. Crystal samples were handled under immersion oil and quickly transferred to a cold nitrogen stream. All measurements were made on a Rigaku Oxford Diffraction Supernova Eos CCD with filtered Cu-Kα radiation at a temperature of 100 K. Using Olex2,[15] the structure was solved with the ShelXT structure solution program using Direct Methods and refined with the ShelXL refinement package[16] using Least Squares minimization.

$Bcp_2PNiPr_2 \cdot BH_3$ (**1**)
The borohydride hydrogen atoms were located in the difference map and refined without restraint.

$Bcp_2PMe \cdot BH_3$ (**3**·BH_3)
All hydrogen atoms were located in the difference map and refined without restraint. Merohedral twinning was modeled according to the following twin law: (-1 0 0 0 -1 0 0 0 1, BASF = 0.67(1)).

Bcp_2PCyb (**4**)
All hydrogen atoms were located in the difference map and refined without restraint.

BcpXPhos (**5**)
Disorder was modeled over two positions with similarity restraints placed on atomic thermal parameters and bond distances.

$(Bcp_2P)_2Fc$ (**6**)
The structure was refined without additional restraints.

Bcp_4P_2 (**7**)
The model was refined with racemic twinning (0.82(2) to 0.18(2)).

$Bcp_2PH \cdot BH_3$ (**8**)
Hydrogen atoms were located in the difference map and refined without restraint.

$^{Bcp4}PCP \cdot 2BH_3$ (**9**·BH_3)
All hydrogen atoms were located in the difference map and refined without restraint.

Table S3. Crystal data and structure refinement for $Bcp_2PNiPr_2 \cdot BH_3$. (**1**)

Empirical formula	$C_{16}H_{31}BNP$	
Formula weight	279.20	
Temperature	100.00(10) K	
Wavelength	1.54184 Å	
Crystal system	Monoclinic	
Space group	P 1 21/c 1	
Unit cell dimensions	a = 8.17290(10) Å	α = 90°
	b = 15.1750(2) Å	β = 102.393(2)°
	c = 14.1367(2) Å	γ = 90°
Volume	1712.43(4) $Å^3$	
Z	4	
Density (calculated)	1.083 Mg/m^3	
Absorption coefficient	1.297 mm^{-1}	
F(000)	616	
Crystal size	0.26 x 0.16 x 0.1 mm^3	
Theta range for data collection	4.329 to 71.647°.	
Index ranges	-9<=h<=9, -18<=k<=18, -15<=l<=17	
Reflections collected	12444	
Independent reflections	3303 [R(int) = 0.0273]	
Completeness to theta = 67.684°	100.0 %	
Absorption correction	Gaussian	
Max. and min. transmission	1.000 and 0.621	
Refinement method	Full-matrix least-squares on F^2	
Data / restraints / parameters	3303 / 0 / 188	
Goodness-of-fit on F^2	1.044	
Final R indices [I>2sigma(I)]	R1 = 0.0363, wR2 = 0.0897	
R indices (all data)	R1 = 0.0397, wR2 = 0.0923	
Largest diff. peak and hole	0.355 and -0.357 $e/Å^{-3}$	

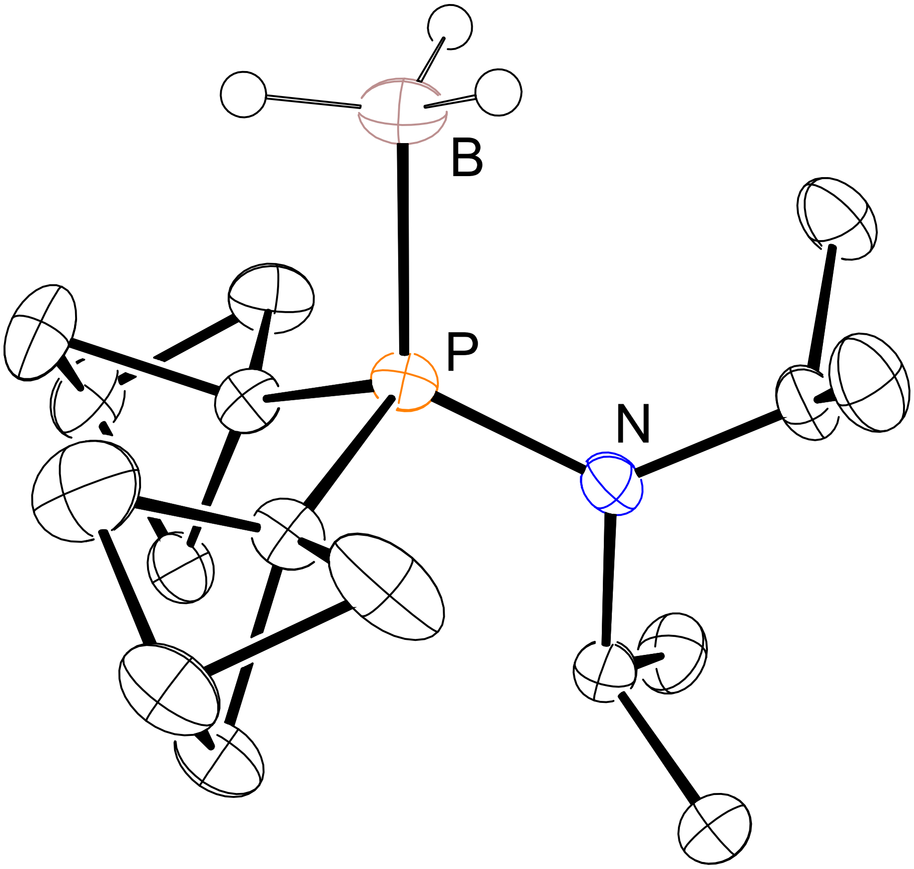

Figure S31. ORTEP of $Bcp_2PNiPr_2{\cdot}BH_3$ (**1**) with ellipsoids shown at 50%.

Table S4. Crystal data and structure refinement for $Bcp_2PMe{\cdot}BH_3$. (**3**·BH_3)

Empirical formula	$C_{11}H_{20}BP$	
Formula weight	194.05	
Temperature	99.9(4) K	
Wavelength	1.54184 Å	
Crystal system	Monoclinic	
Space group	P 1 21/c 1	
Unit cell dimensions	a = 6.39020(10) Å	α = 90°
	b = 13.5878(2) Å	β = 90.3060(10)°
	c = 27.4509(3) Å	γ = 90°
Volume	2383.49(6) $Å^3$	
Z	8	
Density (calculated)	1.082 Mg/m^3	
Absorption coefficient	1.653 mm^{-1}	
F(000)	848	
Crystal size	0.24 x 0.17 x 0.1 mm^3	
Theta range for data collection	3.220 to 71.792°.	
Index ranges	-7<=h<=7, -15<=k<=16, -33<=l<=33	
Reflections collected	27966	
Independent reflections	4633 [R(int) = 0.0384]	
Completeness to theta = 67.684°	100.0 %	
Absorption correction	Gaussian	
Max. and min. transmission	1.000 and 0.631	
Refinement method	Full-matrix least-squares on F^2	
Data / restraints / parameters	4633 / 0 / 396	
Goodness-of-fit on F^2	1.116	
Final R indices [I>2sigma(I)]	R1 = 0.0389, wR2 = 0.0981	
R indices (all data)	R1 = 0.0407, wR2 = 0.1018	
Absolute structure parameter	-0.014(10)	
Largest diff. peak and hole	0.296 and -0.305 e/$Å^{-3}$	

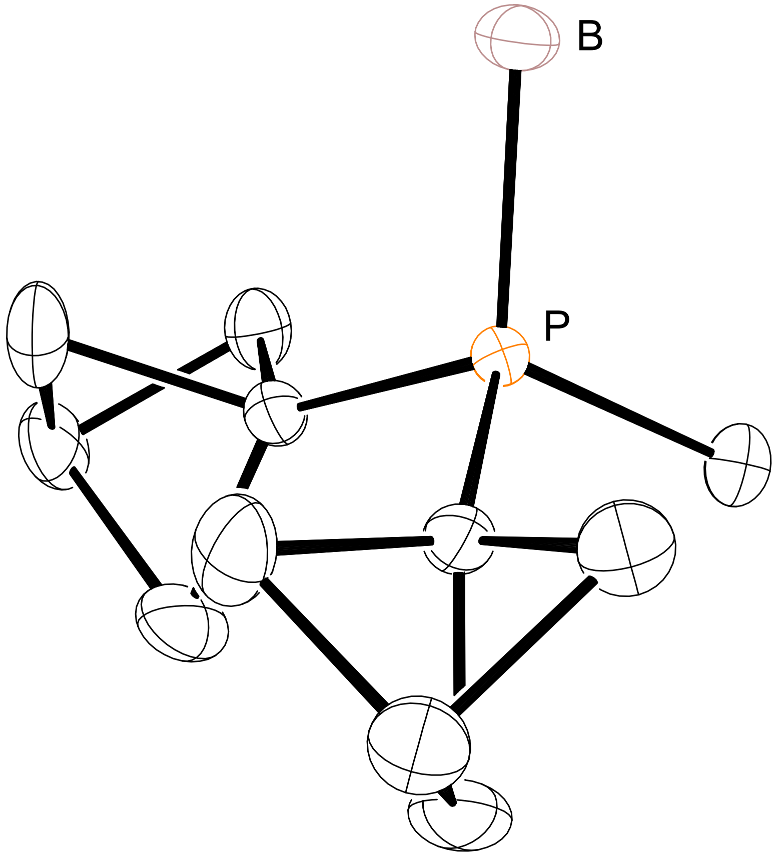

Figure S32. ORTEP of $Bcp_2PMe·BH_3$ (**3**·BH_3) with ellipsoids shown at 50%. One of two molecules in the asymmetric unit omitted for clarity.

Table S5. Crystal data and structure refinement for Bcp_2PCyb. (**4**)

Empirical formula	$C_{14}H_{21}P$	
Formula weight	220.28	
Temperature	100.00(10) K	
Wavelength	1.54184 Å	
Crystal system	Monoclinic	
Space group	P 1 21/c 1	
Unit cell dimensions	a = 13.6647(2) Å	α = 90°
	b = 9.38810(10) Å	β = 107.545(2)°
	c = 10.1621(2) Å	γ = 90°
Volume	1243.01(4) $Å^3$	
Z	4	
Density (calculated)	1.177 Mg/m^3	
Absorption coefficient	1.658 mm^{-1}	
F(000)	480	
Crystal size	0.22 x 0.21 x 0.04 mm^3	
Theta range for data collection	3.392 to 71.717°.	
Index ranges	-16<=h<=16, -11<=k<=11, -10<=l<=12	
Reflections collected	8992	
Independent reflections	2414 [R(int) = 0.0348]	
Completeness to theta = 67.684°	100.0 %	
Absorption correction	Gaussian	
Max. and min. transmission	1.000 and 0.545	
Refinement method	Full-matrix least-squares on F^2	
Data / restraints / parameters	2414 / 0 / 220	
Goodness-of-fit on F^2	1.042	
Final R indices [I>2sigma(I)]	R1 = 0.0323, wR2 = 0.0847	
R indices (all data)	R1 = 0.0370, wR2 = 0.0883	
Absolute structure parameter	-0.014(10)	
Largest diff. peak and hole	0.275 and -0.351 $e/Å^{-3}$	

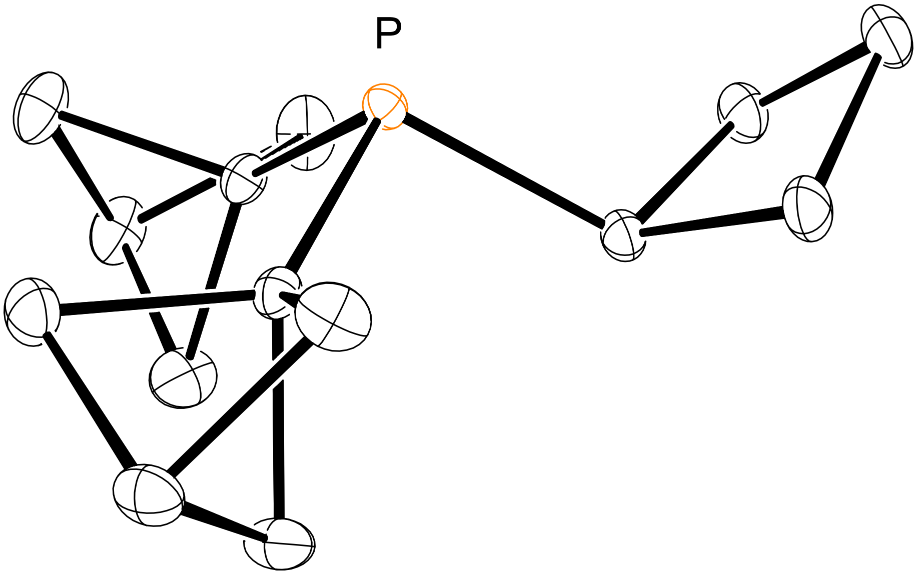

Table S6. ORTEP of Bcp_2PCyb (**4**) with ellipsoids shown at 50%.

Table S7. Crystal data and structure refinement for BcpXPhos. (**5**)

Empirical formula	$C_{31}H_{41}P$	
Formula weight	444.61	
Temperature	100.00(10) K	
Wavelength	1.54184 Å	
Crystal system	Monoclinic	
Space group	P 1 21/c 1	
Unit cell dimensions	a = 16.9579(5) Å	α = 90°
	b = 10.11691(16) Å	β = 117.890(4)°
	c = 17.4273(5) Å	γ = 90°
Volume	2642.58(13) $Å^3$	
Z	4	
Density (calculated)	1.118 Mg/m^3	
Absorption coefficient	1.013 mm^{-1}	
F(000)	968	
Crystal size	0.24 x 0.14 x 0.02 mm^3	
Theta range for data collection	2.948 to 71.819°.	
Index ranges	-19<=h<=20, -12<=k<=9, -20<=l<=21	
Reflections collected	16400	
Independent reflections	5107 [R(int) = 0.0316]	
Completeness to theta = 67.684°	99.9 %	
Absorption correction	Gaussian	
Max. and min. transmission	1.000 and 0.777	
Refinement method	Full-matrix least-squares on F^2	
Data / restraints / parameters	5107 / 73 / 371	
Goodness-of-fit on F^2	1.022	
Final R indices [I>2sigma(I)]	R1 = 0.0544, wR2 = 0.1336	
R indices (all data)	R1 = 0.0617, wR2 = 0.1396	
Absolute structure parameter	-0.014(10)	
Largest diff. peak and hole	0.918 and -0.540 $e/Å^{-3}$	

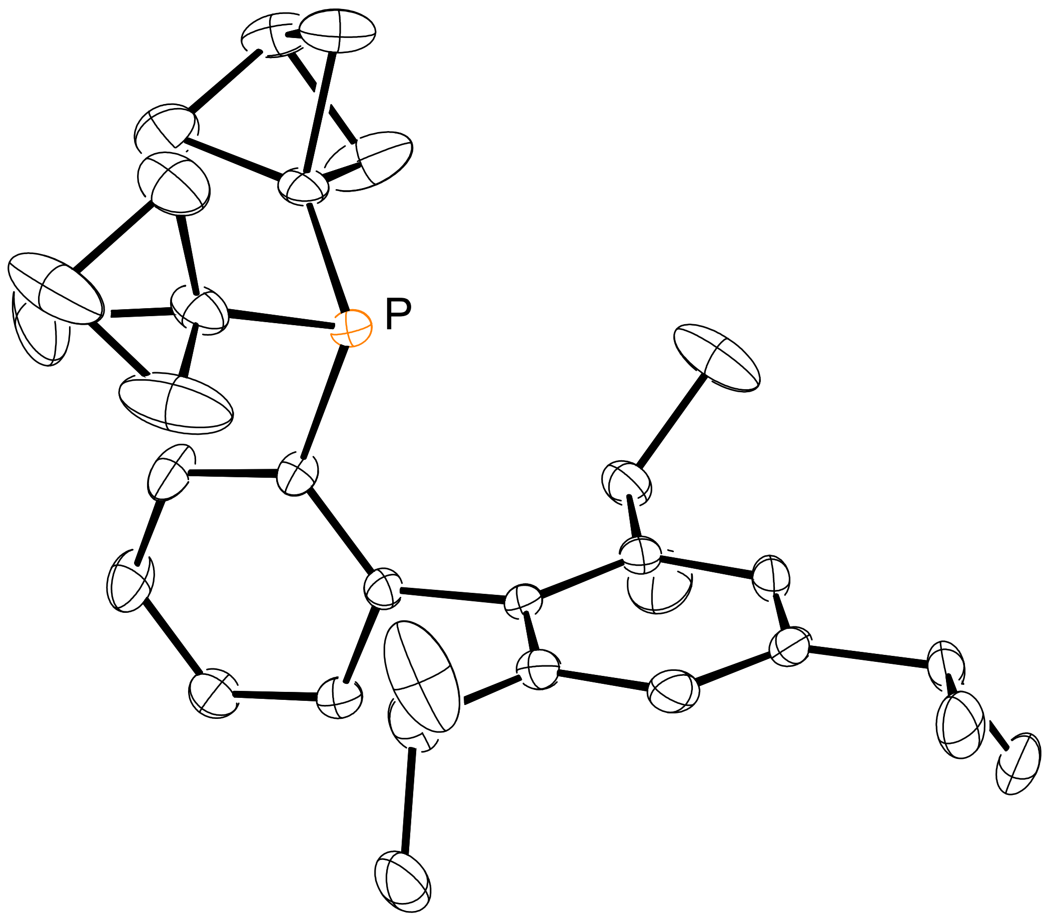

Figure S33. ORTEP of BcpXPhos (**5**) with ellipsoids shown at 50%. Minor disordered components are omitted for clarity.

Figure S34. Crystal data and structure refinement for $(Bcp_2P)_2Fc$. (**6**)

Empirical formula	$C_{30}H_{36}FeP_2$	
Formula weight	514.38	
Temperature	100(1) K	
Wavelength	1.54184 Å	
Crystal system	Monoclinic	
Space group	P 1 21 1	
Unit cell dimensions	a = 6.1273(2) Å	α = 90°
	b = 12.4317(6) Å	β = 91.809(3)°
	c = 17.0737(5) Å	γ = 90°
Volume	1299.90(8) $Å^3$	
Z	2	
Density (calculated)	1.314 Mg/m^3	
Absorption coefficient	5.921 mm^{-1}	
F(000)	544	
Crystal size	0.12 x 0.04 x 0.02 mm^3	
Theta range for data collection	2.589 to 71.807°.	
Index ranges	-7<=h<=6, -12<=k<=15, -21<=l<=17	
Reflections collected	8546	
Independent reflections	4236 [R(int) = 0.0581]	
Completeness to theta = 67.684°	99.9 %	
Absorption correction	Gaussian	
Max. and min. transmission	0.947 and 0.631	
Refinement method	Full-matrix least-squares on F^2	
Data / restraints / parameters	4236 / 1 / 298	
Goodness-of-fit on F^2	1.046	
Final R indices [I>2sigma(I)]	R1 = 0.0639, wR2 = 0.1553	
R indices (all data)	R1 = 0.0738, wR2 = 0.1614	
Absolute structure parameter	-0.014(10)	
Largest diff. peak and hole	1.067 and -0.464 e/$Å^{-3}$	

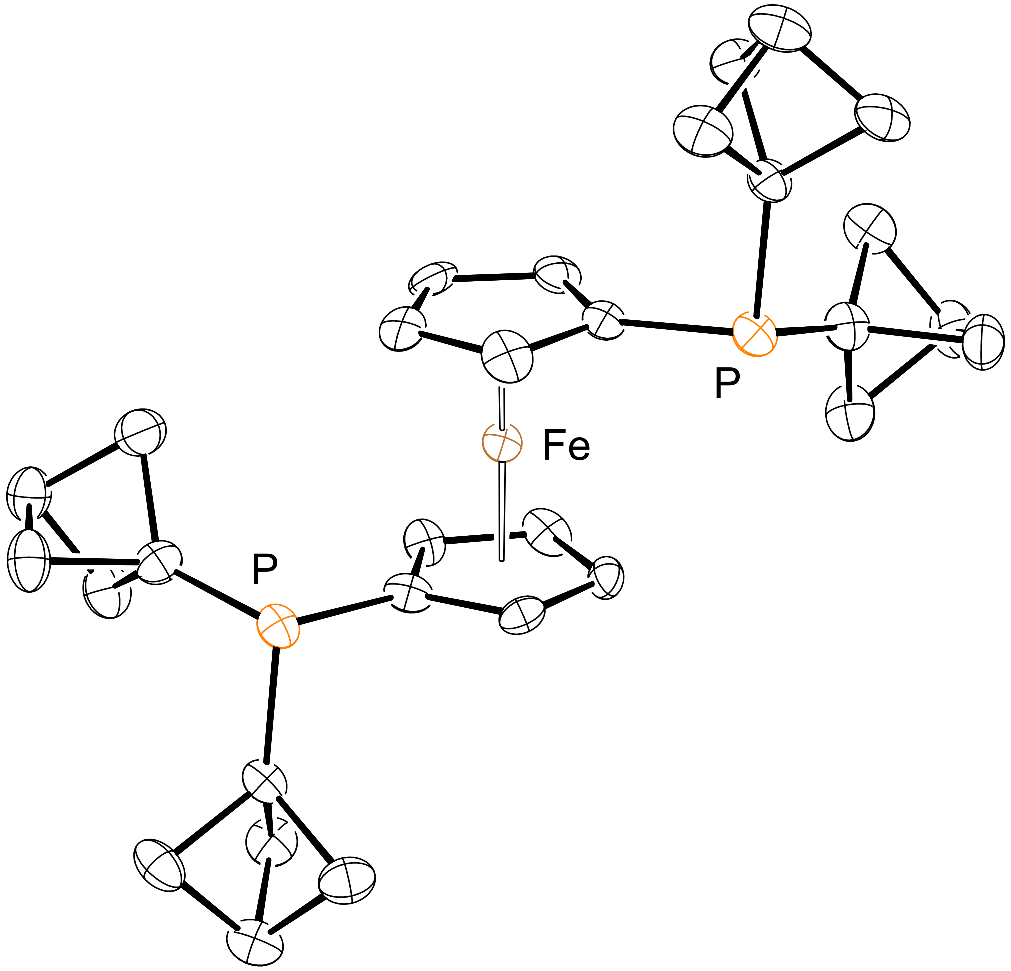

Figure S35. ORTEP of $(Bcp_2P)_2Fc$ (**6**) with ellipsoids shown at 50%

Table S8. Crystal data and structure refinement for Bcp_4P_2. (**7**)

Empirical formula	$C_{20}H_{28}P_2$	
Formula weight	330.36	
Temperature	99.91(19) K	
Wavelength	1.54184 Å	
Crystal system	Orthorhombic	
Space group	$P2_12_12_1$	
Unit cell dimensions	a = 9.10860(10) Å	α = 90°
	b = 10.26990(10) Å	β = 90°
	c = 19.5606(2) Å	γ = 90°
Volume	1829.79(3) $Å^3$	
Z	4	
Density (calculated)	1.199 Mg/m^3	
Absorption coefficient	2.093 mm^{-1}	
F(000)	712	
Crystal size	0.34 x 0.1 x 0.07 mm^3	
Theta range for data collection	4.521 to 71.744°.	
Index ranges	-11<=h<=10, -12<=k<=11, -23<=l<=24	
Reflections collected	8987	
Independent reflections	3396 [R(int) = 0.0291]	
Completeness to theta = 67.684°	100.0 %	
Absorption correction	Gaussian	
Max. and min. transmission	1.000 and 0.512	
Refinement method	Full-matrix least-squares on F^2	
Data / restraints / parameters	3396 / 0 / 200	
Goodness-of-fit on F^2	1.020	
Final R indices [I>2sigma(I)]	R1 = 0.0256, wR2 = 0.0618	
R indices (all data)	R1 = 0.0267, wR2 = 0.0627	
Absolute structure parameter	0.18(2)	
Largest diff. peak and hole	0.230 and -0.170 $e/Å^{-3}$	

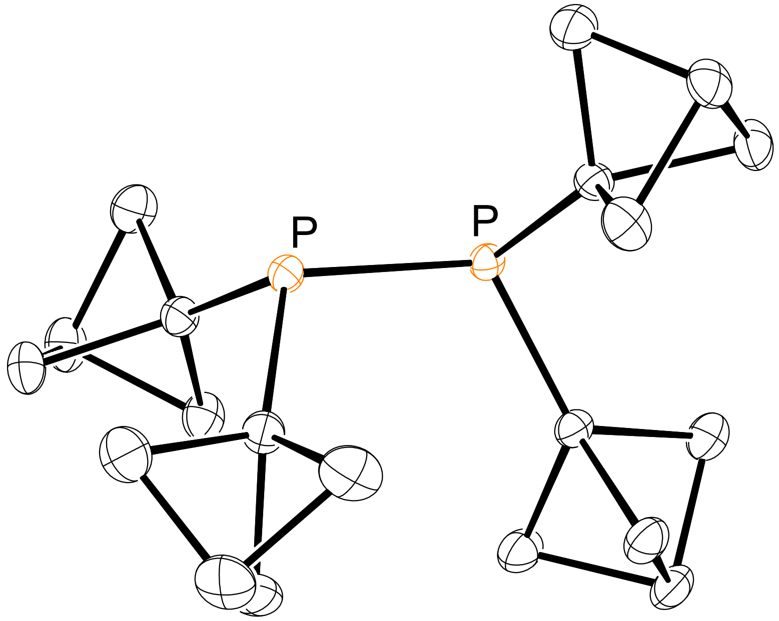

Figure S36. ORTEP of Bcp_4P_2 (**7**) with ellipsoids shown at 50%.

Table S9. Crystal data and structure refinement for $Bcp_2PH{\cdot}BH_3$. (**8**)

Empirical formula	$C_{10}H_{18}BP$	
Formula weight	180.02	
Temperature	100.01(10) K	
Wavelength	1.54184 Å	
Crystal system	Monoclinic	
Space group	P 1 21/n 1	
Unit cell dimensions	a = 5.84270(10) Å	α = 90°
	b = 13.81710(10) Å	β = 98.1580(10)°
	c = 13.7357(2) Å	γ = 90°
Volume	1097.65(3) $Å^3$	
Z	4	
Density (calculated)	1.089 Mg/m^3	
Absorption coefficient	1.761 mm^{-1}	
F(000)	392	
Crystal size	0.21 x 0.11 x 0.1 mm^3	
Theta range for data collection	4.563 to 71.784°.	
Index ranges	-6<=h<=5, -16<=k<=16, -16<=l<=15	
Reflections collected	8253	
Independent reflections	2098 [R(int) = 0.0250]	
Completeness to theta = 67.684°	99.8 %	
Absorption correction	Gaussian	
Max. and min. transmission	1.000 and 0.742	
Refinement method	Full-matrix least-squares on F^2	
Data / restraints / parameters	2098 / 0 / 181	
Goodness-of-fit on F^2	1.042	
Final R indices [I>2sigma(I)]	R1 = 0.0289, wR2 = 0.0724	
R indices (all data)	R1 = 0.0304, wR2 = 0.0738	
Absolute structure parameter	0.18(2)	
Largest diff. peak and hole	0.354 and -0.224 $e/Å^{-3}$	

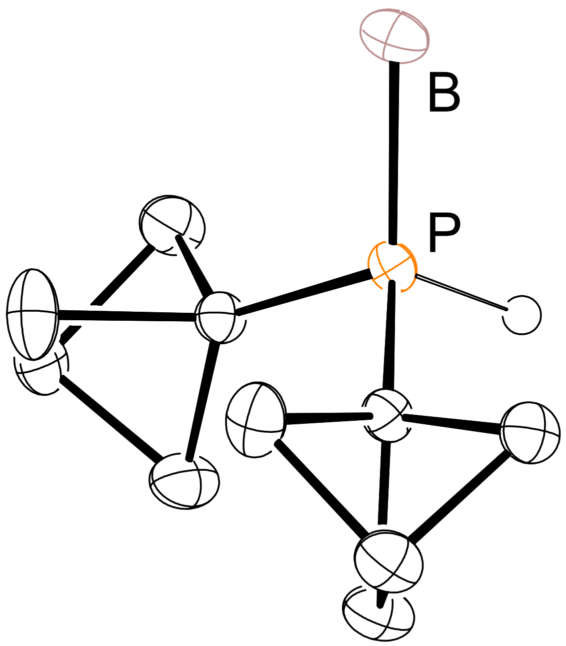

Figure S37. ORTEP of $Bcp_2PH{\cdot}BH_3$ (**8**) with ellipsoids shown at 50%.

Table S10. Crystal data and structure refinement for $^{Bcp4}PCP\cdot 2BH_3$. (**9**·$2BH_3$)

Empirical formula	$C_{28}H_{42}B_2P_2$	
Formula weight	462.17	
Temperature	100.4(8) K	
Wavelength	1.54184 Å	
Crystal system	Triclinic	
Space group	P-1	
Unit cell dimensions	a = 8.2150(2) Å	α = 91.663(2)°
	b = 12.9678(3) Å	β = 101.656(2)°
	c = 13.4786(3) Å	γ = 102.196(2)°
Volume	1370.67(6) $Å^3$	
Z	2	
Density (calculated)	1.120 Mg/m^3	
Absorption coefficient	1.516 mm^{-1}	
F(000)	500	
Crystal size	0.33 x 0.2 x 0.16 mm^3	
Theta range for data collection	3.357 to 71.838°.	
Index ranges	-10<=h<=9, -15<=k<=15, -16<=l<=16	
Reflections collected	20208	
Independent reflections	5298 [R(int) = 0.0228]	
Completeness to theta = 67.684°	100.0 %	
Absorption correction	Gaussian	
Max. and min. transmission	1.000 and 0.409	
Refinement method	Full-matrix least-squares on F^2	
Data / restraints / parameters	5298 / 0 / 457	
Goodness-of-fit on F^2	1.034	
Final R indices [I>2sigma(I)]	R1 = 0.0288, wR2 = 0.0758	
R indices (all data)	R1 = 0.0296, wR2 = 0.0765	
Extinction coefficient	n/a	
Largest diff. peak and hole	0.263 and -0.293 e/$Å^{-3}$	

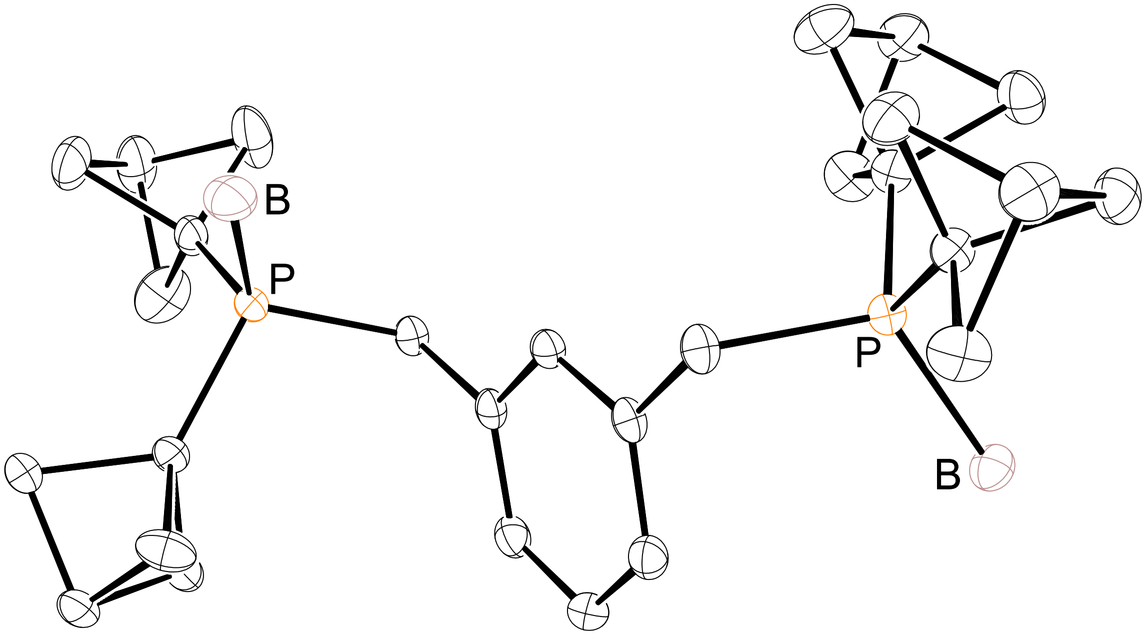

Figure S38. ORTEP of ^{Bcp4}PCP·2BH$_3$ (**9**·BH$_3$) with ellipsoids shown at 50%.

VI. References

(1) de Jong, G. B.; Ortega, N.; Lutz, M.; Lammertsma, K.; Slootweg, J. C., Easy Access to Phosphine-Borane Building Blocks. *Chem. Eur. J.* **2020**, *26*, 15944-15952.

(2) Bär, R. M.; Kirschner, S.; Nieger, M.; Bräse, S., Alkyl and Aryl Thiol Addition to [1.1.1]Propellane: Scope and Limitations of a Fast Conjugation Reaction. *Chem. Eur. J.* **2018**, *24*, 1373-1382.

(3) Gianatassio, R.; Lopchuk, J. M.; Wang, J.; Pan, C.-M.; Malins, L. R.; Prieto, L.; Brandt, T. A.; Collins, M. R.; Gallego, G. M.; Sach, N. W.; Spangler, J. E.; Zhu, H.; Zhu, J.; Baran, P. S., Strain-release amination. *Science* **2016**, *351*, 241-246.

(4) Lichtscheidl, A. G.; Ng, V. W. L.; Müller, P.; Takase, M. K.; Schrock, R. R., Molybdenum Monoaryloxide Pyrrolide Alkylidene Complexes That Contain Mono-ortho-substituted Phenyl Imido Ligands. *Organometallics* **2012**, *31*, 2388-2394.

(5) *Gaussian 16, Revision B.01*, M. J. Frisch, G. W. T., H. B. Schlegel, G. E. Scuseria, M. A. Robb, J. R. Cheeseman, G. Scalmani, V. Barone, G. A. Petersson, H. Nakatsuji, X. Li, M. Caricato, A. V. Marenich, J. Bloino, B. G. Janesko, R. Gomperts, B. Mennucci, H. P. Hratchian, J. V. Ortiz, A. F. Izmaylov, J. L. Sonnenberg, D. Williams-Young, F. Ding, F. Lipparini, F. Egidi, J. Goings, B. Peng, A. Petrone, T. Henderson, D. Ranasinghe, V. G. Zakrzewski, J. Gao, N. Rega, G. Zheng, W. Liang, M. Hada, M. Ehara, K. Toyota, R. Fukuda, J. Hasegawa, M. Ishida, T. Nakajima, Y. Honda, O. Kitao, H. Nakai, T. Vreven, K. Throssell, J. A. Montgomery, Jr., J. E. Peralta, F. Ogliaro, M. J. Bearpark, J. J. Heyd, E. N. Brothers, K. N. Kudin, V. N. Staroverov, T. A. Keith, R. Kobayashi, J. Normand, K. Raghavachari, A. P. Rendell, J. C. Burant, S. S. Iyengar, J. Tomasi, M. Cossi, J. M. Millam, M. Klene, C. Adamo, R. Cammi, J. W. Ochterski, R. L. Martin, K. Morokuma, O. Farkas, J. B. Foresman, and D. J. Fox, Gaussian, Inc., Wallingford CT, 2016.

(6) Gusev, D. G., Donor Properties of a Series of Two-Electron Ligands. *Organometallics* **2009**, *28*, 763-770.

(7) Adamo, C.; Barone, V., Exchange functionals with improved long-range behavior and adiabatic connection methods without adjustable parameters: The mPW and mPW1PW models. *The Journal of Chemical Physics* **1998**, *108*, 664-675.

(8) Perdew, J. P.; Burke, K.; Wang, Y., Generalized gradient approximation for the exchange-correlation hole of a many-electron system. *Physical Review B* **1996**, *54*, 16533-16539.

(9) Burke, K.; Perdew, J. P.; Wang, Y., Derivation of a Generalized Gradient Approximation: The PW91 Density Functional. In *Electronic Density Functional Theory: Recent Progress and New Directions*, Dobson, J. F.; Vignale, G.; Das, M. P., Eds. Springer US: Boston, MA, 1998; pp 81-111.

(10) Bilbrey, J. A.; Kazez, A. H.; Locklin, J.; Allen, W. D., Exact ligand cone angles. *J. Comput. Chem.* **2013**, *34*, 1189-1197.

(11) Tolman, C. A., Steric effects of phosphorus ligands in organometallic chemistry and homogeneous catalysis. *Chem. Rev.* **1977**, *77*, 313-348.
(12) Mansson, R. A.; Welsh, A. H.; Fey, N.; Orpen, A. G., Statistical Modeling of a Ligand Knowledge Base. *Journal of Chemical Information and Modeling* **2006**, *46*, 2591-2600.
(13) Perry, G. L.; Schley, N. D., Tris(bicyclo[1.1.1]pentyl)phosphine: An Exceptionally Small Tri-tert-alkylphosphine and Its Bis-Ligated Pd(0) Complex. *J. Am. Chem. Soc.* **2023**, *145*, 7005-7010.
(14) Newman-Stonebraker, S. H.; Smith, S. R.; Borowski, J. E.; Peters, E.; Gensch, T.; Johnson, H. C.; Sigman, M. S.; Doyle, A. G., Univariate classification of phosphine ligation state and reactivity in cross-coupling catalysis. *Science* **2021**, *374*, 301-308.
(15) Dolomanov, O. V.; Bourhis, L. J.; Gildea, R. J.; Howard, J. A. K.; Puschmann, H., OLEX2: a complete structure solution, refinement and analysis program. *J. Appl. Crystallogr.* **2009**, *42*, 339-341.
(16) Sheldrick, G., A short history of SHELX. *Acta Crystallogr. Sect. A* **2008**, *64*, 112-122.
